# Magnetosome Gene Duplication as an Important Driver in the Evolution of Magnetotaxis in the *Alphaproteobacteria*

**DOI:** 10.1128/mSystems.00315-19

**Published:** 2019-10-29

**Authors:** Haijian Du, Wenyan Zhang, Wensi Zhang, Weijia Zhang, Hongmiao Pan, Yongxin Pan, Dennis A. Bazylinski, Long-Fei Wu, Tian Xiao, Wei Lin

**Affiliations:** a Key Laboratory for Marine Ecology and Environmental Sciences, Institute of Oceanology, Chinese Academy of Sciences, Qingdao, China; b Laboratory for Marine Ecology and Environmental Science, Qingdao National Laboratory for Marine Science and Technology, Qingdao, China; c Key Laboratory of Earth and Planetary Physics, Institute of Geology and Geophysics, Chinese Academy of Sciences, Beijing, China; d Innovation Academy for Earth Science, Chinese Academy of Sciences, Beijing, China; e France-China Joint Laboratory for Evolution and Development of Magnetotactic Multicellular Organisms, Chinese Academy of Sciences, Beijing, China; f College of Earth Sciences, University of Chinese Academy of Sciences, Beijing, China; g Laboratory of Deep-sea Microbial Cell Biology, Institute of Deep-sea Science and Engineering, Chinese Academy of Sciences, Sanya, China; h International Associated Laboratory of Evolution and Development of Magnetotactic Multicellular Organisms, CNRS-Marseille/CAS-Beijing-Qingdao-Sanya, Marseille, France; i CAS Key Laboratory for Experimental Study under Deep-sea Extreme Conditions, Institute of Deep-sea Science and Engineering, Chinese Academy of Sciences, Sanya, China; j School of Life Sciences, University of Nevada at Las Vegas, Las Vegas, Nevada, USA; k Aix-Marseille University, CNRS, LCB, Marseille, France; Ghent University

**Keywords:** *Terasakiella*, evolution, gene duplication, genomes, magnetosome gene cluster, magnetotactic bacteria, magnetotaxis, pure cultivation

## Abstract

The evolution of microbial magnetoreception (or magnetotaxis) is of great interest in the fields of microbiology, evolutionary biology, biophysics, geomicrobiology, and geochemistry. Current genomic data from magnetotactic bacteria (MTB), the only prokaryotes known to be capable of sensing the Earth’s geomagnetic field, suggests an ancient origin of magnetotaxis in the domain *Bacteria*. Vertical inheritance, followed by multiple independent magnetosome gene cluster loss, is considered to be one of the major forces that drove the evolution of magnetotaxis at or above the class or phylum level, although the evolutionary trajectories at lower taxonomic ranks (e.g., within the class level) remain largely unstudied. Here we report the isolation, cultivation, and sequencing of a novel magnetotactic spirillum belonging to the genus *Terasakiella* (*Terasakiella* sp. strain SH-1) within the class *Alphaproteobacteria*. The complete genome sequence of *Terasakiella* sp. strain SH-1 revealed an unexpected duplication event of magnetosome genes within the *mamAB* operon, a group of genes essential for magnetosome biomineralization and magnetotaxis. Intriguingly, further comparative genomic analysis suggests that the duplication of *mamAB* genes is a common feature in the genomes of alphaproteobacterial MTB. Taken together, with the additional finding that gene duplication appears to have also occurred in some magnetotactic members of the Deltaproteobacteria, our results indicate that gene duplication plays an important role in the evolution of magnetotaxis in the *Alphaproteobacteria* and perhaps the domain *Bacteria*.

**IMPORTANCE** A diversity of organisms can sense the geomagnetic field for the purpose of navigation. Magnetotactic bacteria are the most primitive magnetism-sensing organisms known thus far and represent an excellent model system for the study of the origin, evolution, and mechanism of microbial magnetoreception (or magnetotaxis). The present study is the first report focused on magnetosome gene cluster duplication in the *Alphaproteobacteria*, which suggests the important role of gene duplication in the evolution of magnetotaxis in the *Alphaproteobacteria* and perhaps the domain *Bacteria*. A novel scenario for the evolution of magnetotaxis in the *Alphaproteobacteria* is proposed and may provide new insights into evolution of magnetoreception of higher species.

## Abstract (precis)

A diversity of organisms can sense the geomagnetic field for the purpose of navigation. Magnetotactic bacteria are the most primitive magnetism-sensing organisms known thus far and represent an excellent model system for the study of the origin, evolution, and mechanism of microbial magnetoreception (or magnetotaxis). The present study is the first report focused on magnetosome gene cluster duplication in the *Alphaproteobacteria*, which suggests the important role of gene duplication in the evolution of magnetotaxis in the *Alphaproteobacteria* and perhaps the domain *Bacteria*. A novel scenario for the evolution of magnetotaxis in the *Alphaproteobacteria* is proposed and may provide new insights into evolution of magnetoreception of higher species.

## INTRODUCTION

Many organisms sense the Earth’s geomagnetic field in some way and use its direction and/or intensity for navigation and migration over both short and long distances (1). This behavior, termed magnetoreception, is widespread among various phyla of the domains *Bacteria* and *Eukarya*. However, the origin and evolution of magnetoreception as well as the underlying mechanisms involved remain poorly understood. Magnetotactic bacteria (MTB), a phylogenetically and physiologically diverse group of prokaryotes that biomineralize intracellular, membrane-bounded, magnetic iron crystals (magnetosomes) composed of magnetite (Fe_3_O_4_) and/or greigite (Fe_3_S_4_), are characterized by their ability to sense and swim along geomagnetic field lines, a behavior recognized as magnetotaxis or microbial magnetoreception (2). In addition to the well-known occurrence of magnetoreception in animals, including insects, fishes, birds, and mammals, MTB represent an excellent model system for studies of the origin and evolution of magnetoreception, as prokaryotic microorganisms are the earliest life forms that evolved on Earth (3).

MTB are phylogenetically diverse and have thus far been identified in phyla of the domain *Bacteria*. These include the *Proteobacteria*, *Nitrospirae*, and *Planctomycetes* phyla and the candidate phyla of *Omnitrophica* (previously known as candidate division OP3) and *Latescibacteria* (previously known as candidate division WS3) (3–6). The genes responsible for magnetosome biomineralization and microbial magnetoreception are clustered in MTB genomes (referred to as magnetosome gene clusters [MGCs]) (6). Some genes within MGCs are conserved in all known MTB genomes over a broad taxonomic range, providing great insights into the evolutionary history of magnetotaxis. Recent genomic and phylogenetic studies have suggested an ancient origin of magnetotaxis, involving lineage-specific evolution in prokaryotes of the domain *Bacteria* (7). At or above the class or phylum level, vertical inheritance, followed by multiple independent MGC loss, is considered to be one of the major forces that drove the evolution of magnetotaxis (7–10). However, the subsequent evolutionary trajectories of MGCs within different bacterial classes appear to be much more complicated and less understood (11).

The recent rapid expansion of the number of MTB isolated in pure culture and genomes from the *Alphaproteobacteria* makes this class suitable for investigating the evolution of magnetotaxis at lower taxonomic levels. MGCs of the *Alphaproteobacteria* are often organized into several operons (e.g., *mamAB*, *mamGFDC*, *mamXY*, and *mms6* operons); the *mamAB* operon contains several core genes that are essential for magnetosome formation and arrangement (12, 13). Horizontal gene transfer (HGT) is considered to have some roles in shaping the evolution of magnetotactic *Alphaproteobacteria*. For instance, a genomic region termed the magnetosome islet (MIS), which is thought to have been acquired through HGT, containing several magnetosome genes outside the MGC was identified in the genome of Magnetospirillum magneticum strain AMB-1 (14), and some proteins (e.g., MamK) within MIS and MCG are expected to interact with each other (15). More recently, a comparison of phylogenetic trees of the region encoding magnetosome proteins of representative alphaproteobacterial MTB suggests that either ancient HGT or ancient duplication events may have occurred during the evolution of magnetotaxis in this class (16). In the present study, we report the isolation of a novel magnetotactic alphaproteobacterium whose genome contains two copies of the *mamAB* operon. Together with a comprehensive analysis of alphaproteobacterial MGCs, our results suggest that magnetosome gene duplication is an important driver in the evolution of magnetotaxis in the *Alphaproteobacteria*.

## RESULTS AND DISCUSSION

**FIG 1:**
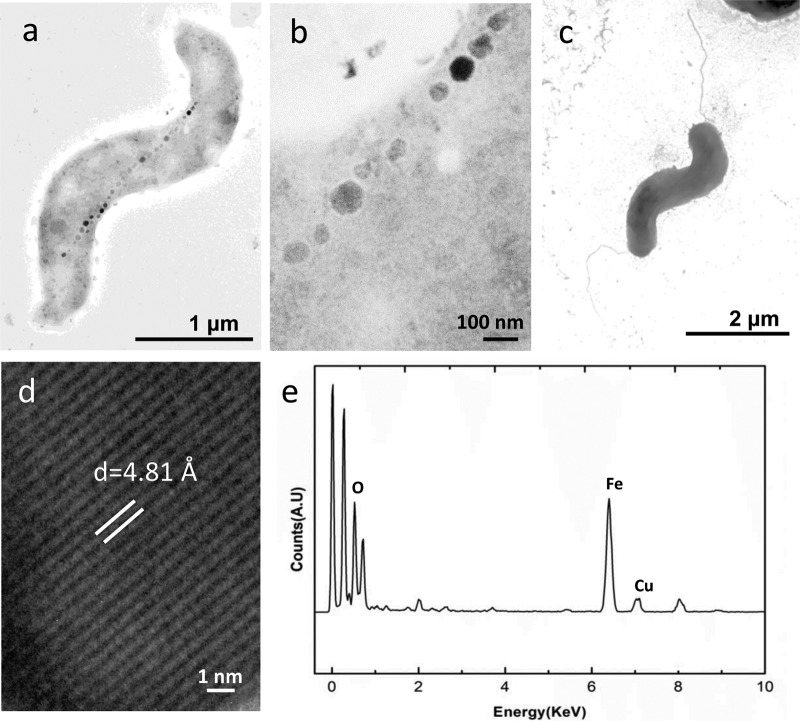
Cell morphology and magnetosomes of *Terasakiella* sp. strain SH-1. (a to c) Transmission electron microscopy (TEM) images showing cellular morphology of strain SH-1 (a), magnetosomes in a chain (b), and flagella of strain SH-1 (c). (d) High-resolution TEM image of magnetosomes. d, distance. (e) Energy-dispersive X-ray spectroscopy of magnetosomes. Counts are shown in arbitrary units (A.U).

A novel magnetotactic spirillum belonging to the genus *Terasakiella* (*Terasakiella* sp. strain SH-1) was isolated in pure culture, and its complete genome was sequenced. Briefly, sediment samples were collected from the intertidal zone of “the remotest corners of the globe” (Ultima Thule) in Sanya, China (18°17′29″N, 109°20′59″E). MTB were magnetically enriched and concentrated and then inoculated into a semisolid growth medium modified from that of *Magnetospira* sp. strain QH-2 (17). Cells of strain SH-1 were vibrioid to helicoid with a single flagellum at each pole (Fig. 1a to c). Cells contained 5 to 19 magnetosomes, with crystals with an average length and width of 48.3 ± 8.9 nm and 35.7 ± 5.2 nm, respectively (*n* = 22) (Fig. 1b). Energy-dispersive X-ray spectroscopy showed that the magnetosome crystals consisted of elongated, prismatic Fe_3_O_4_ (Fig. 1d and e).

**FIG 2:**
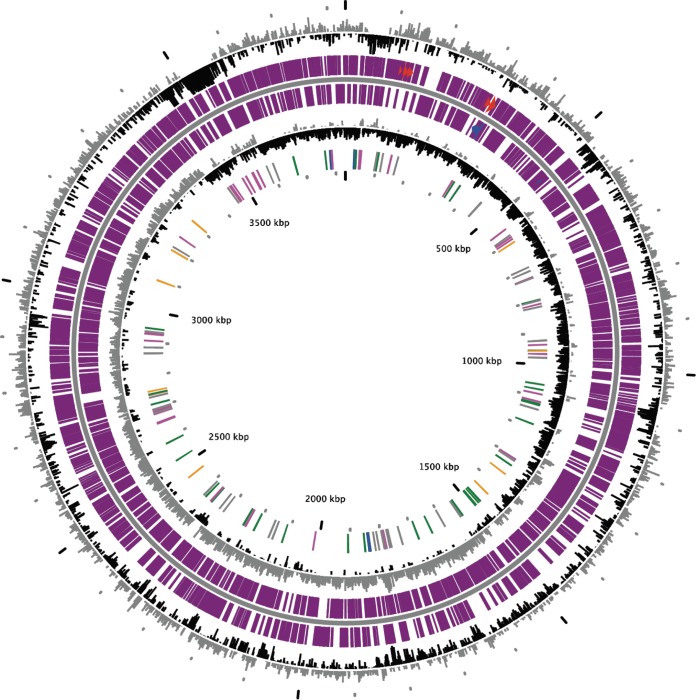
Circular diagrams of the chromosome of *Terasakiella* sp. strain SH-1. The outermost circle (circle 1) shows GC percent deviation in a 1,000-bp window. The next circle, circle 2, shows predicted CDSs transcribed in the clockwise direction. The next circle, circle 3, shows predicted CDSs transcribed in the counterclockwise direction. Circle 4 shows GC skew (G+C/G-C) in a 1,000-bp window. The innermost circle, circle 5, shows rRNA (blue), tRNA (green), miscellaneous RNA (orange), transposable elements (pink), and pseudogenes (gray). The genes in circles 2 and 3 are color coded as follows: red and blue indicate MicroScope-validated annotation, orange indicates MicroScope automatic annotation with a reference genome, and purple indicates primary/automatic annotations.

**FIG 3:**
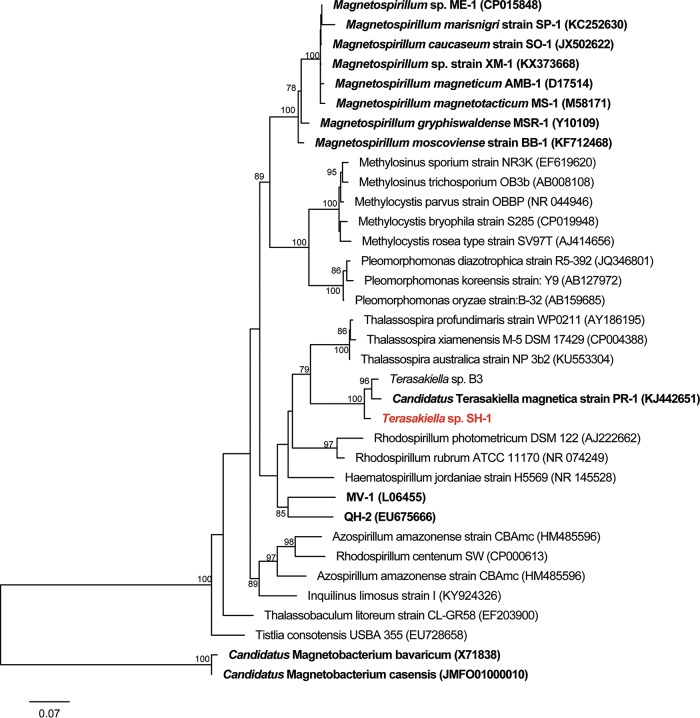
Phylogenetic analysis of *Terasakiella* sp. strain SH-1. Maximum-likelihood phylogenetic tree based on 16S rRNA gene sequences. “*Candidatus* Magnetobacterium bavaricum” and “*Candidatus* Magnetobacterium casensis” were used as the outgroup. *Terasakiella* sp. strain SH-1 isolated in this study is marked in red. Previously reported MTB are shown in boldface type.

The genome of strain SH-1 comprises a single 3,832,570-bp circular chromosome (Fig. 2) with a G+C content of 47.5%. The chromosome contains 3,633 predicted coding sequences (CDSs), including 50 tRNAs and three copies of rRNA operon (5S, 16S, and 23S). The 16S rRNA gene sequence of SH-1 is 96.7% identical to that of *Candidatus* Terasakiella magnetica strain PR-1 (16) and the average amino acid identity (AAI) between strains SH-1 and PR-1 is 80.5%. Consequently, SH-1 represents a new species in the genus *Terasakiella* in the *Alphaproteobacteria* (Fig. 3).

**FIG 4:**
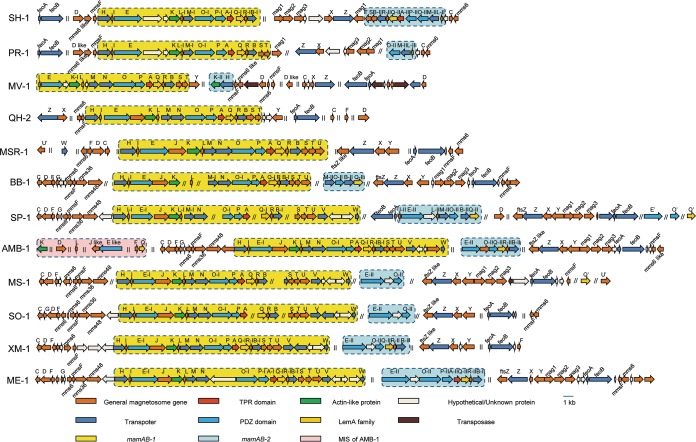
Arrangement of MGCs from representative MTB belonging to the *Alphaproteobacteria.* The yellow and blue regions represent two copies of the *mamAB* operon (referred to as *mamAB-1* and *mamAB-2*, respectively). The pink region in Magnetospirillum magneticum strain AMB-1 represents the magnetotaxis islet (MIS) previously identified (14). The gene names with apostrophes represent the potential paralogous magnetosome genes scattered outside the MGCs. The intervals made up of genes not related to magnetosome genes (ǁ) and the gaps between different contigs (*∥*) are indicated.

The MGC of strain SH-1 includes a 42,440-bp genomic region consisting of 47 genes, which unexpectedly, contains two copies of the *mamAB* operon in reverse order (Fig. 4). One copy (yellow region in Fig. 4) contains *mamH*, *mamI*, *mamE*, *mamK*, *mamL-I*, *mamM-I*, *mamO-I*, *mamP-I*, *mamA-I*, *mamQ-I*, *mamR-I*, and *mamB-I*, while another copy (blue region in Fig. 4) contains *mamT*, *mamS*, *mamB-II*, *mamR-II*, *mamQ-II*, *mamA-II*, *mamP-II*, *mamO-II*, *mamM-II*, and *mamL-II*. These apparent gene operon duplications are separated by a 172,254-bp region containing 145 CDSs that appear to not be related to known magnetosome genes. A BLASTp search revealed that magnetosome proteins of MamL, -M, -O, -P, -A, -Q, -R, and -B are perfectly duplicated (100% identity) except for MamO (47.8% identity). To avoid any sequencing or assembly artifacts, the accuracy of the genomic DNA sequence of the two *mamAB* operons was further checked and confirmed through PCR-based sequencing (see Table S1 and Data Set S1 in the supplemental material). The PCR products of *mamAB-1* and *mamAB-2* are 100.0% and 99.9% identical to their templates, respectively, proving that the two *mamAB* operons really exist.

10.1128/mSystems.00315-19.1 TABLE S1 The primers designed for *mamAB-1* and *mamAB-2*. Download Table S1, DOCX file, 0.03 MB. Copyright © 2019 Du et al. 2019 Du et al. This content is distributed under the terms of the Creative Commons Attribution 4.0 International license.

10.1128/mSystems.00315-19.3 DATA SET S1 The regions involved in PCR-based sequencing. The boldface regions represent sequences of *mamAB-1* and *mamAB-2*. The underlined sequences are targeted by primers. Download Data Set S1, PDF file, 0.2 MB. Copyright © 2019 Du et al. 2019 Du et al. This content is distributed under the terms of the Creative Commons Attribution 4.0 International license.

**FIG 5:**
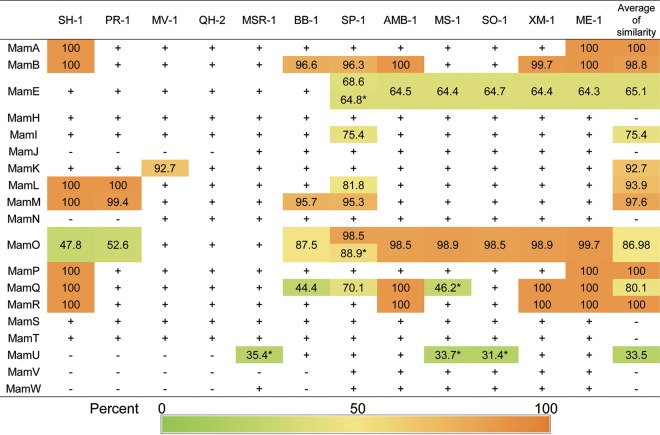
Sequence identities of paralogous magnetosome proteins in the *mamAB* operon. Sequence identities (shown as percentages) were calculated using “BLAST and Pattern Search” of the MicroScope platform (27). A plus symbol indicates that the protein was detected in the genome without a paralog. A minus symbol indicates that the protein was not detected in the genome. The similarities related to MIS in *Magnetospirillum magneticum* strain AMB-1 are not shown here. Numbers with an asterisk superscript indicate potential paralogous magnetosome genes scattered outside the MGCs. All similarities show the identities between the query sequence and the genes in *mamAB*-*1*. The strains are shown at the top of the figure and are *Terasakiella* sp. strain SH-1, *Candidatus* Terasakiella magnetica strain PR-1, Magnetovibrio blakemorei strain MV-1, *Magnetospira* sp. strain QH-2, Magnetospirillum gryphiswaldense strain MSR-1, *Ms. moscoviense* BB-1, *Ms. marisnigri* SP-1, *Ms. magneticum* strain AMB-1, *Ms. magnetotacticum* MS-1, *Ms. caucaseum* SO-1, *Magnetospirillum* sp. strain XM-1, and *Magnetospirillum* sp. strain ME-1.

In order to identify whether magnetosome gene operon duplication is a common event in the alphaproteobacterial MTB, we further investigated and compared the MGCs of 12 representative MTB from the *Alphaproteobacteria* (including *Candidatus* Terasakiella magnetica strain PR-1, Magnetovibrio blakemorei strain MV-1, *Magnetospira* sp. strain QH-2, Magnetospirillum gryphiswaldense (*Ms. gryphiswaldense*) strain MSR-1, Ms. moscoviense strain BB-1, *Ms. marisnigri* strain SP-1, *Ms. magneticum* strain AMB-1, *Ms. magnetotacticum* strain MS-1, *Ms. caucaseum* strain SO-1, *Magnetospirillum* sp. strain XM-1, *Magnetospirillum* sp. strain ME-1, and *Terasakiella* sp. strain SH-1). We noted apparent duplication events of *mamAB* operons in all analyzed genomes except *Magnetospira* sp. strain QH-2 and *Ms. gryphiswaldense* strain MSR-1 (Fig. 4 and 5). Some potentially duplicated magnetosome genes have been identified in the genomes of strains MSR-1, SP-1, MS-1, and SO-1, which, however, scatter outside MGCs (Fig. 4 and 5). Of the 19 important *mam* genes within the *mamAB* operon (Fig. 5), 12 have more than one copy in the same genome, including *mamA*, *mamB*, *mamI*, *mamE*, *mamK*, *mamL*, *mamM*, *mamO*, *mamP*, *mamQ*, *mamR*, and *mamU*. The proteins of MamA, MamB, MamK, MamL, MamM, MamO, MamP, MamQ, and MamR represent high level of identities (>80%) to their corresponding paralogs. Inverted duplications of *mamAB* operons were identified in *Terasakiella* sp. SH-1, *Candidatus* Terasakiella magnetica strain PR-1, and Magnetovibrio blakemorei strain MV-1. The two copies of the *mamAB* operon (designated AB-1 [yellow region in Fig. 4] and AB-2 [blue region in Fig. 4]) appear to be discontinuous and are separated by an approximately 6- to 172-kb interval (7 to 145 CDSs) or distributed in different contigs. For each *mamAB* operon of *Terasakiella* sp. SH-1, *Candidatus* Terasakiella magnetica strain PR-1, *Magnetospirillum* sp. strain ME-1, *Magnetospirillum* sp. strain XM-1, and *Magnetospirillum magneticum* strain AMB-1, most genes in *mamAB-1* and *mamAB-2* (except for *mamE* and *mamO*) represent high levels of similarity (>98%) (Fig. 5). In addition to the *mamAB* operon, multiple copies of genes within the *mms6* operon are also identified in some genomes, which, however, have low levels of sequence identity (Fig. 4 and Table S2). Previous studies have reported the duplications of *mamQ*, *mamR*, and *mamB* within the MGC of *Magnetospirillum magneticum* strain AMB-1 (12, 18) and the duplications of *mamE* and *mamO* exist in multiple lineages of MTB (19), while the present study suggests that the duplication event of magnetosome genes is very common in the *Alphaproteobacteria*.

10.1128/mSystems.00315-19.2 TABLE S2 Sequence identities (as percentages) of potential paralogous magnetosome proteins in the *mms6* operon. A minus symbol indicates that the protein was detected in the genome without a paralog. Download Table S2, DOCX file, 0.03 MB. Copyright © 2019 Du et al. 2019 Du et al. This content is distributed under the terms of the Creative Commons Attribution 4.0 International license.

**FIG 6:**
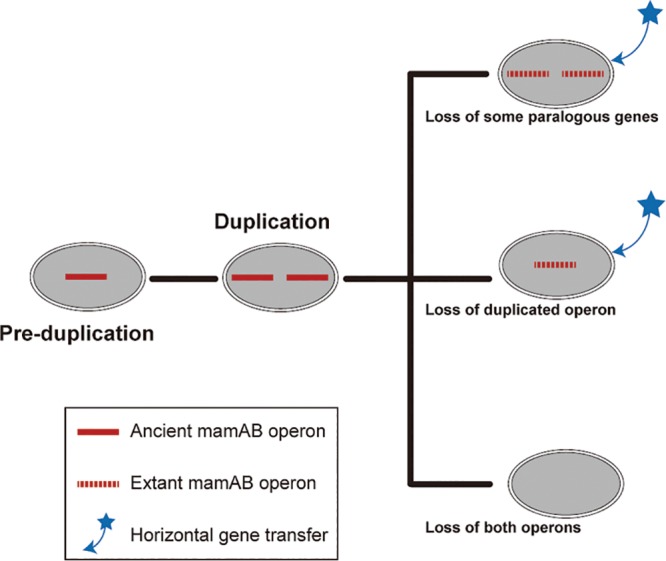
Proposed scenario for the evolution of the *mamAB* operon in the *Alphaproteobacteria*. The ancient *mamAB* operon might be duplicated in the ancestor of the *Alphaproteobacteria*. Multiple instances of loss of paralogous genes or of entire operon(s) occurred during evolution, resulting in extant patchy distribution of MTB. Some magnetosome genes or gene operons might be acquired through horizontal gene transfer as previously suggested (14, 16). A few lineages might lose the whole duplicated operon and many other populations retain both operons with loss events of different paralogous genes. It would seem that the most common outcome of all these gene rearrangements is the loss of both operons and results in non-MTB.

The persistence of various paralogous magnetosome *mamAB* genes in the large majority of *Alphaproteobacteria* MTB identified here clearly suggests that gene duplication is an important force driving the evolution of magnetotaxis in this class. The duplication of a long magnetosome gene operon containing up to eight genes in *Terasakiella* sp. strain SH-1 has not been previously observed in the *Alphaproteobacteria*, leading us to propose an entire *mamAB* operon duplication event in the ancestor of *Alphaproteobacteria* (Fig. 6). During subsequent evolution, massive gene or operon loss occurred, with a few lineages losing most, if not all, genes in a single operon (e.g., *Ms. gryphiswaldense* strain MSR-1 and *Magnetospira* sp. strain QH-2) and many other populations retaining both operons with loss events of different paralogous genes (e.g., *Terasakiella* sp. SH-1, *Candidatus* Terasakiella magnetica strain PR-1, *Magnetospirillum magneticum* strain AMB-1, and *Magnetospirillum* sp. strain ME-1). It would seem that the most common outcome of all these gene rearrangements is the loss of both operons and results in non-MTB (Fig. 6).

Our results raise an interesting question: although gene duplication has been recognized in the genomes of prokaryotes for many years, why were magnetosome genes specifically duplicated during evolution? Previous studies suggest that some duplicated magnetosome genes are functionally redundant (12) or work with paralogues as polymers (15, 18). Considering the generally high sequence identities between paralogous magnetosome genes (Fig. 5), we suggest that the magnetosome gene duplications in the magnetotactic *Alphaproteobacteria* are due to selection for increased gene dosage or for functional buffering. Magnetotaxis is recognized to efficiently guide cells of MTB to their preferred microenvironments in aquatic habitats (20). In addition, magnetosome crystals in some MTB have been experimentally shown to perform enzyme-like activities in the elimination of toxic intracellular reactive oxygen species (21). Thus, both magnetotaxis and magnetosome crystals appear to offer fitness advantages for the survival of MTB in nature. Considering that the *mamAB* operon is essential for magnetosome biomineralization and magnetotaxis (12, 22), the presence of duplicated *mamAB* genes could increase genetic robustness and buffer the magnetotaxis and magnetosome biomineralization functions, especially considering the relatively high frequency of spontaneous loss of magnetosome genes in some MTB strains (e.g., *Magnetospirillum*) (11). The fact that the retention of a paralogous gene is biased with regard to the essential *mam* genes (e.g., *mamB*, *mamE*, *mamL*, *mamM*, *mamO*, and *mamQ*) for magnetosome biomineralization also supports this hypothesis (Fig. 5).

Gene duplication provides the opportunity for acquiring new genes and creating genetic novelty through the divergence between duplicated genes (neofunctionalization or subfunctionalization) (23). A previous study has suggested that the duplication and neofunctionalization and/or new gene acquisition could explain the presence of multiple proteases (MamE and MamO) in MTB belonging to the classes of the *Alphaproteobacteria*, *Gammaproteobacteria*, and Deltaproteobacteria (19). Moreover, it has been proposed that the MGC involved in Fe_3_S_4_ biomineralization originated from the duplication of the Fe_3_O_4_-type MGC with subsequent divergence that may have occurred in the Deltaproteobacteria (3). More recently, it has been suggested that, as an alternative scenario, duplication and divergence of ancient MGC might have occurred much earlier, e.g., in the last common ancestor of all extant MTB, which generated both Fe_3_O_4_- and Fe_3_S_4_-type MGCs (7). Duplicated magnetosome genes in the *Alphaproteobacteria* could have led to these genes evolving novel functions, and these paralogous genes might or will differentiate in some aspects of their functions, which could help MTB to better adapt to changing environments.

In conclusion, results from this study support the idea that gene duplication, followed by gene loss and divergence of the *mamAB* operon, is an important process that shaped the evolution of magnetotaxis in the *Alphaproteobacteria* and perhaps even throughout the domain *Bacteria*. The discovery of novel MTB species (e.g., *Terasakiella* sp. strain SH-1) with duplicated magnetosome genes also reinforces the need to further explore the genomic diversity of environmental MTB.

## MATERIALS AND METHODS

### Isolation and cultivation of strain SH-1.

MTB were concentrated magnetically by attaching the south pole of a permanent magnet (0.05 T) outside a bottle containing water and sediment about 1 cm above the sediment surface for 30 min and then separated from nonmagnetotactic bacteria using the magnetic racetrack technique (24). These separated MTB cells were inoculated into the modified semisolid medium. The modified semisolid medium consisted of an artificial seawater base, containing the following (per liter): 19.45 g NaCl, 5.92 g MgCl_2_ · 6H_2_O, 3.24 g Na_2_SO_4_, 0.55 g KCl, 1.8 g CaCl_2_, 5 ml modified Wolfe’s mineral elixir (25), 0.3 g NH_4_Cl, 2.38 g HEPES, 0.5 g peptone, and 0.5 g agar. The pH was adjusted to 7.7. The medium was then autoclaved, followed by the addition of neutralized 4 ml of 10% l-cysteine · HCl · H_2_O solution, 20 ml of 25% (wt/vol) aqueous sodium thiosulfate, 0.5 ml vitamin solution (25), 2.35 ml of 8% NaHCO_3_, and 2 ml of 0.01 M ferric quinate. The cultures were incubated at 25°C until a microaerophilic band of cells was observed at the oxic/anoxic interface (OAI). Liquid 2216E medium (Hopebio, Qingdao, China) was used for the mass culture of strain SH-1 after it was isolated. The liquid 2216E medium consisted of the following (per liter): 19.45 g NaCl, 5.98 g MgCl_2_ · 6H_2_O, 3.24 g Na_2_SO_ 4_, 1.8 g CaCl_2_, 0.55 g KCl, 0.08 g KBr, 0.034 g SrCl, 0.022 g H_3_BO_3_, 0.004 g Na_2_SiO_3_, 0.0024 g NaF, 0.0016 g NH_4_NO_3_, 0.008 g Na_2_HPO_4_, 40 ml of 0.01 M ferric citrate, 5 g peptone, and 1 g yeast extract. To obtain a pure culture of strain SH-1, separate colonies were obtained using solid 2216E medium (15 g agar per liter). Colonies were removed aseptically, and the process was repeated three times.

### Optical and electron microscopy observations.

A 30-μl sample was removed to prepare a hanging drop (26) for microscopic examination using optical microscopy (Olympus BX51 equipped with a DP71 camera system; Olympus, Tokyo, Japan). For transmission electron microscopy (TEM), 2 μl of a liquid culture of strain SH-1 was deposited on a Formvar-coated copper grid (EMCN, Beijing, China) and allowed to sit for 3 to 5 min; the grid was then washed with distilled water and air dried. Cells for the detection of flagella were stained with 1% uranyl acetate for 1 min. For TEM a Hitachi H8100 transmission electron microscope operated at 75 kV was used. Magnetosomes were analyzed by high-resolution transmission electron microscopy (HRTEM) using a JEM2100 transmission electron microscope operated at 200 kV and equipped for energy-dispersive X-ray spectroscopy (EDXS). The length and width of magnetosome crystals were measured using images imported into Adobe Photoshop.

### 16S rRNA phylogenetic analysis, genome sequencing, and comparative analysis of genes.

Freshly grown cells were washed three times with sterile distilled water and freeze-thawed three times by freezing in liquid nitrogen and thawing at 100°C. Amplification of the 16S rRNA gene was achieved by PCR in an Eppendorf Mastercycler, using the universal bacterial primers 27F (5′-AGAGTTTGATCCTGGCTCAG-3′) and 1492R (5′-GGTTACCTTGTTACGACTT-3′) (Sangon Biotech, Shanghai, China). The PCR products were purified, cloned into pMD18-T vectors (TaKaRa, Dalian, China), and transformed into competent Escherichia coli Top10 cells (Tiangen Biotech, Beijing, China). Several clones were sequenced and validated the clonal population of strain SH-1.

The genome of strain SH-1 was sequenced using a PacBio RS II platform and Illumina HiSeq 4000 platform at the Beijing Genomics Institute (BGI) (Shenzhen, China). The circular diagrams of SH-1 chromosome, gene prediction, and sequence identity of paralogous magnetosome proteins were calculated using the MicroScope platform (27). AAI values were estimated with the calculator of the enveomics collection (28).

16S rRNA gene sequences for phylogenetic analyses were obtained from the GenBank database. The sequences were aligned using the SINA (29). Gblocks was used to eliminate poorly aligned and noisy portions of the alignment (30). A phylogenetic tree was constructed using IQ-TREE v1.5.5 through ultrafast bootstrap (-bb 100000) (31). 16S rRNA gene sequences from “*Candidatus* Magnetobacterium bavaricum” and “*Candidatus* Magnetobacterium casensis” were used as the outgroup.

The accuracy of the genomic DNA sequence of the two *mamAB* operons was checked by PCR. A total of 26 and 17 pairs of primers were designed for *mamAB-1* and *mamAB-2*, respectively (see Table S1 in the supplemental material). The regions that primers targeted are shown in Data Set S1 in the supplemental material.

### Data availability.

The genome sequence was deposited in GenBank and carries the BioProject accession number PRJNA529092.
