# Supplementary material for: Magnetosome Gene Duplication as an Important Driver in the Evolution of Magnetotaxis in the Alphaproteobacteria
Source: mSystems. 2019 Oct 29;4(5):e00315-19. doi: 10.1128/mSystems.00315-19 (PMC6819731; doi:10.1128/mSystems.00315-19)
Supplement: TABLE S1 [file mSystems.00315-19-st001.docx]

| primers for *mamAB-1* | Sequence(5'→3') |
| --- | --- |
| A1F | TTTGTATAAGGCGCAGCTTG |
| A1R | CAACCGCTTCAATCAGTTCT |
| A2F | TTGATGAAGCCAATGCCAAG |
| A2R | CAAGGCTGTAATCTGCTCGT |
| A3F | GTCTTCCGGTTTTCGTACTG |
| A3R | TAACACCCCACAAGAACATA |
| A4F | CGTCATATCCGCACCAAGTT |
| A4R | TAAACGACACAGAACGCAGG |
| A5F | GTGTGCGTTTCCAGTCTTGC |
| A5R | GACCTGACCAAAACAGCCTT |
| A6F | TCCGTCATCAAAAAGGTCCC |
| A6R | GCAAAGCCTTTCAAACGGGT |
| A7F | CAGGGCCAGCTTTTCGACCA |
| A7R | ACAGCTTTCTCCGGCCAAAT |
| A8F | TCTGAGGAAATCACACACGC |
| A8R | CCCAAAAAGCCAGTTGCAAG |
| A9F | GCGCAAAACCAGATTCGTGA |
| A9R | CACCAAGACCGAGGCCGAGA |
| A10F | AAGGTTTGATGATCGGTAAGAC |
| A10R | CACCAGCTTTTGCCGTTG |
| A11F | TGAAATGGAACGCGAAGGTA |
| A11R | GCAGATCGCCGACAGGAAAT |
| A12F | GTCATGTTTTCTCTGATCGG |
| A12R | GACAATTTCCCACTCAAACG |
| A13F | TGTTCATCGGGCTGTTCCTG |
| A13R | CCAGCTTGACCCATCGGCAT |
| A14F | TGGCACTGGGCAATGACACT |
| A14R | TCGGTGGGCCTTGACGCATC |
| A15F | GTTCGTACTTTCTTGCTCGA |
| A15R | TGCGCAAGGTTGGTGTGTTC |
| A16F | CGTCGTGTGAATTCTCCGTT |
| A16R | ATTAAAGGCATCCATCAAAA |
| A17F | CAACAGTGAAGTCTACAACG |
| A17R | TTCAACTAAGACTTCATCCT |
| A18F | CGATTATCTGAACGTCTGGC |
| A18R | TTTGACATCACCGCAGTATA |
| A19F | TCGACGCTGCTGTTAGTGGG |
| A19R | AGCGCAAATAACAAGATAGC |
| A20F | CTTTGGCGATGTGCAGGTGT |
| A20R | GGCCTCATCGACGGTGCAGT |
| A21F | AAGATTCTGTCATGGGCCTG |
| A21R | GTCACTTGGGCGTTATTACC |
| A22F | CGGTGTCAACGTTGCCTTTA |
| A22R | TCAGGAAGATGGAAGGCAGA |
| A23F | CATTGGTTGCCTTTATCCAC |
| A23R | GCGAATACCCTTAACCACAG |
| A24F | CGATGATCCTGCCCGGTGAT |
| A24R | AAGTCTTTGCGCCGTTGTAA |
| A25F | TGCAACGGCTCAAACGCTCC |
| A25R | AACATGGTCACGATAGAGGC |
| A26F | AGCAGCATAATTGAGTATTT |
| A26R | TCATATGAAAACACTAGAGG |
|  |  |
| primers for *mamAB-2* | Sequence(5'→3') |
| B1F | CACTAGCCCTGCGGAGTC |
| B1R | ACCCAGCTCCATCGGCCA |
| B2F | TGATGTGCGCGGCCAAAC |
| B2R | GGCGCTCAATCACGGCTC |
| B3F | TGATCTGCCGGATTTGGAAA |
| B3R | GATGACACACTTGCTAACTC |
| B4F | AACAGATTTTACGTGAGCAA |
| B4R | ATAGCCCCGCGCACAACTGA |
| B5F | TGACTTCCTTGACCTGTTGC |
| B5R | TCCCGTTATCGACCAGAGCC |
| B6F | GTCTTTACGTTTCCCGCCCA |
| B6R | CAATAGACAAAGAGGCACCA |
| B7F | TTCCCTATGGTGTATTTCCG |
| B7R | TAGCCCAGAAGGATCGCAAT |
| B8F | CGGTAACATCGCTCAAGTCT |
| B8R | AACACATTGGCCGAACTGGT |
| B9F | GGTACCACGCACTTCGGTCT |
| B9R | CATCACTTGTCGCCTACGCA |
| B10F | AGGAGAACCCACAGACGATC |
| B10R | GCGGGTATGTCTGTTGTGGT |
| B11F | CTCAATCCCGATGCGTGCCA |
| B11R | CTGGACCTTAATGACATGGG |
| B12F | CGGGAAGTTGGTGAAATCCG |
| B12R | TTCCACGACCTGTTTACCAT |
| B13F | CAGATGCAGCATGACTTCGC |
| B13R | AAAGCACAGAACACTATGGG |
| B14F | TTGAGGCGCAGGAGAAGCAA |
| B14R | GCGTACAACCGAACAACTGA |
| B15F | CATCATGTTTGTATCCGTTA |
| B15R | AAATACGATAAATGCGGTAA |
| B16F | AATGACGAGAAGAGAGCTGA |
| B16R | GAAACGCCATGATTCCTACA |
| B17F | GCCTGAATTCTATTGCGAGA |
| B17R | ATCATTCACCTCATGCCCTT |
