# Supplementary material for: Magnetosome Gene Duplication as an Important Driver in the Evolution of Magnetotaxis in the Alphaproteobacteria
Source: mSystems. 2019 Oct 29;4(5):e00315-19. doi: 10.1128/mSystems.00315-19 (PMC6819731; doi:10.1128/mSystems.00315-19)
Supplement: DATA SET S1 [file mSystems.00315-19-sd001.pdf]

>*mamAB-1* (bold region: SH1_v1_0119 ~ SH1_v1_0144)

TTTGTATAAGGCGCAGCTTGACTCAGTTTCAAAATAATTAAAAAGGTGCATGTAAAGCAATTAAAGG
TGGTTCGATTTCTACGTTAATCGGTGCAATTTATCTTTTTGGGTAAAAAAGTATATTTCAGAGGTATA
ATATAAGGCCGTATGCGCAAGCTCTTATGCTAATTATTAGTATCATTATAATATAATGAAGTAAAAG
AGGGAATCTACGTAGTTGACAGTGCAAGTAAGTCGCAATACGATGCGAAAATTGAATACCTACATAT
GTATTCAATAGGGGAAACTGCTTAAAGTTTGGAAGGTAAGAT**ATGGCGACTTTAGCAAATCTGGG**
AAAAGGCCAGCGGGCCTATATCAATCAAATCGGGGGCGATTCAACTCTGAAGCGTAAGCTCTT
GTCTATGGGGATTGTCAAAGGCGTTGAAGTGTCTTTGTCTCACACGGCCCCATTGGGTGATCC
ACGCATCTATTCTTTGCTGGGTTATGATCTGTCTCTTCGCAATGACGAAGCGGCCAATATCACC
GTTGACAGCACAAAGCCTTAATCAAGGCATTCGGAGTTACATTTCGTGAACACACAAACCAAG
ACCTTGACGGTTGCCTTGGCGGGGAACCCGAACTGTGGTAAAACGACCCTGTTGAATAAACTG
ACAGGCTCTCATAAAGGCGTGGGTAACTATGCCCGTGTGACAATTGACAAACAGGGCAGCAAG
CTGACCCATAAAGGCTGGACGATCAATGTGATTGATCTGCCGGGGATTTATTCTTTAAGCTCCC
AGTCCCCGGAAGAAATCGTTGGCCGTGATTTTATTCAGGACGAACAGCCGGATCTCATCCTGA
ACGTTCTGGATGCAACAAACATTCAGCGTAGCCTGTTTTTGTCCACCCAATTGATTGAAATGGG
TGTGCCGCGCATTTACGACATCAACATGATTGATGAAGCCAATGCCAAGGATATCTCTTTTGAC
ACCCTCGCGCTGGCTGAAATCCTCGGCAGCCCGGTGGTGGAGACAGAAGCGCGCAAAGGTTTT
GGTTTTGAACTGCTGCTCGACACCATTGTTGAGATGGCGGAAAAAGGTCTGCCCAAAGACAGC
ATTAACATCAAATATGACAGCCACCTTGAAGAAGCCATCGTTGATGTACAGAAAATCATTTCTG
ACCTTCACCCTGGGGAGATGAATGAACATCAGACCCGTTGGTTGGCAATCAAGCTGATGGAAG
GCGATGATGACCTGCTTAAACGTGAAGCAGAACATGCAGAACTGATTGAAGCGGTTGATGCCA
AACGTGCAGAGATCGAACGCACCCATGGTGAAAGCACGGAAGTGATGTTTGCACAAGGGCGTT
ACGGTTTCATCAACGGTGTTGTTCAAGAAGCGGTGACTTTGCCTGCAACGTCTGAACAATCTTC
AAGCCTGACACAAAAACTGGATCACATCTTCTTGCATCGTTTCCTCGGCCTGCCGATTTTCCTT
GGCCTGATTTGGGCCATGTTTGAAGCAACCTTTACATTGGGGACTTACCCGATGGACTGGATT
GATGGCCTGATGGGTGATTTCACCGATGTTGTTGCAGGCGCCATGAGCGAAGGCTTGCTGCGT
GACCTGATGGTTGACGGTATCTTGGCCGGGGTTGGGGGCACGATCATCTTCCTGCCCAACATT
GTGATCCTGTTCTTCTTTATGGCTGTGTTCAGCGAAACCGGTTATCTCGCCCGTTCTGCCTTTT
TGGTGGATCGCACCATGCATGCCTTTGGTCTGCACGGGAAAGCCATGATCCCGCTGGTGATGG
GCTTTGGCTGTAACGTTCCCGCCATTATGGCGTGTCGCACGATCGAAAGCCCGCGTGCACGCC
TGATCGCCATTTTGGTGAACCCGTTCATGGCTTGTACAGCGCGTCTTCCGGTTTTCGTACTGTT
TGCCGGGGCCTTCTTTGCCGATGTCGCAGGGACCATGGTTTTTGCCATGTATATGCTGAGTATC
GTTGTGGCCATGCTGTCGTCGATCTTCTTATCTAAATTTATTATCAAAGGGCAAAACGAGTCCT
TTGTTATGGAATTGCCGCCGTTCCGCGTACCCACATTCAGCGGCATTTTCTTCCATATGTTTGA
AAAGGCCATGGGCTTTATCAAGAAAGTTGGTGGTATCATCCTTGTTGGTTCGATCATCATCTGG
ATCTTGCAAGCCTTCCCGCAGAATGTTGAATATTCTCAGGATTTTGAAGCGAACATCGAAGCGC

TTCAGGCCCAGCCGGAAACAGAGGCTCGTAACGAGCAGATTACAGCCTTGTCACGCCAGCAGA
CGCTGGAAACAATGGAAAAAAGCTATCTGGGTCAAATCGGTGTAGCCGTAACCCCCGTGTTTG
AGCCGCTTGGCTTTAACTGGAAAGACACCGTTGCGATCCTGACTGGCGTTGTTGCCAAAGAGG
TTGTGGTTGCCACATATGCCGTGCTTTACGGTCAGGATGAGGAATCTACTGAAGAATCCGTCTC
CCTTCGTGAAGCCATGACGGGCATTATGACGCCGCTTGTTGCCTTTACCTTCATGGTCTTTGCG
CTTCTTTATGCGCCGTGCTTCAGCACATTGGCGGTGATCAAGCGTGAAGCCGGTGGCTGGAAA
TGGGTAGGCTTCTCTGTTGCCTTCTCCGTTACCGTTGCCTGGTCTTTGGCTTTCGTTATTTCGA
TTATCGGGAATATATTGATCTGATAATTAGACAGGCTTAAAAAAGGATCGTCGTGTGATTATTT
ACACCTCGATCCTTTTTTTGCTAATATCGCTTAAATTGAGACTTATTCTCATATCAAAAGGTAGT
GACAATGGAAGCTTTAATCCTCACCATCATTTTTGCCGTCTGTGTTTTTCTGGCGATGCGTCAT
ATCCGCACCAAGTTTAATCCCAGTGATGGCTGTGGCAGTGGTTGCGGGGGCTGTAGCTCTCAG
CCGACCTGCAGCACGACTGAAAAAAAATAATCAAGAAGATCAAAAATGGACCTGCTTGGTAAT
ACGCTTCACCTCTATCTAGAGGCTGCACCCTGGTTGTTATTCGGCCTGATTATGGCCGGTGTGA
TCAAAGCCTGGATGTCTGAAAAGGCCGTGCAAAAATATGTCGGCGGGCGGGGTATTGGGTCCA
TTTTCGGCGCAGCCTTGTTTGGGGCGCCTTTACCTTTATGTTCTTGTGGGGTGTTACCCGCAGC
CGTCGGTATGCGCCGTGCAGGCGGCTCGCGCCCCGCCACGCTGTCTTTCTTGATCTCTACTCC
GGAAACCGGGGTTGATTCTGTGGCCGTATCCTACGCTTTGCTGGGGCCGTTCATGGCAATTGT
GCGCCCGATTGCTGCGGTAAGCAGCGCGGTCTTTACCGGGCTTTTGTCTTTGTTCCTTCCCGAA
GAAGATCAAAAGCCTACGCCAGCCGCAGCACCATCGAGCTGCTGTACCAGCACCTGTTGCGGG
TCTTCCAAGCCTGTTGAGCCGAAAAAGCCCAATGCCTTTGTCAAAACCTATGACGGAATTGCGT
ATGCACTCAGCGATATTCTGGATGATATCGCCCTGTGGCTTGGCATCGGCTTGCTGATTGCCG
GTGTTGTCACCACCTATGTACCGGAACAGGCGCTGGTGGAATGGGGCAGCGGCCCGCTGGCC
ATGTTGGTCATGCTGGTGGTTGGAATTCCCATGTATATTTGCGCTACAGCTTCCACCCCTTTGG
CAGCCAGTTTTTTGTTGGCGGGTGTGTCGCCCGGCGCCGTGATGGTCTTTTTACTGGCTGGTC
CGGCAACCAACATGGCAACCATTGCGGTTGTACGCAACGAAATGGGCACCCGCACCATGTGGG
TGTATCTGACAGGTGTGTGCGTTTCCAGTCTTGCCATTGGCTTTATCGTCAACCAATTGGTGAA
CCTGTGGGCCATTGATATTCAGGCTGAACTTGCAGCCAGTGCCCATGTTCTACCCGCCGGAAT
GGAAGAGGCGTGCGGTATTTTGCTGGCCTTGCTGTTTGTGAAACGCATCCCTGCCATTGTCAG
TGCGAAGCTGGCCCCGAGTAACGGCTGACCCTTATCCCCACGGCCTGCGTTCTGTGTCGTTTA
AGCGATAAAGCGGCAGGCGCGACAGGGTTAAAATCAGGAAAAACGCCACATCGACTTCCTGAT
CATCCAAGGCATGATGGATTTGCTTGCGCAAGGCCGCATCCAGATTATCCCCATACGTTTGGCT
GAGTGTTGCCAGATAATCCTGCGGTGTGGTTGTGCGCCACAGGCTGGCCCCGCTTTTGCGTTC
ATCTGCGGTGGATTTGGTGGGGTCCGTATGGTCGCGCAGGGCGTCGATAATACGGTTAATGAA
GATGTCGTAATCGTTGTTTTGGTCCAGATGCATGTCGCTTTTCCTTTTTAAAACTCCATCTCTG
GTCACCAGAGTTTTTTCTGGTGACCGGGAGTTTAGCAACATGCTGTTAGACATGCGGACCTATT
TCCCGAGGGTGTTATATTCAGCCCACTCCCGACCATAGGAAAAGCCTTGCATGCCGTTTTACAG

ACACACAAAGACCGCCCAGTCGGGGGCAGTAAACCGCTAACAGAACGTTGCTAAACGTCATCT
TCACCATAAGGTGAAAAACCGTGGGAACAAGGGGGGATTGGAAAAAGGTCCAAACCATCTGT
CGTCATCCCGGATTTATTCCGGGACAATCCCGATGGGCCAGAGATTGCCTTTCCAAGGTTGTC
GCGCAACAGGTGCGCGATGACGGGGATGAGGCGCGTCATCCCCAGCTTGACTGGGGATCTTT
GTCCGTCATCAAAAAGGTCCCCGCATCCGCGAGGACGACAGGTGGGGGATGGGGGGGATGAT
CGGGCAACAAAAAACCGCCAGCAGACTGTTCTGAACTGGCGGTGTTTCGTGTGTGGGTTTTTC
AGCTTTTAGGACATGGCCTTAAAAGGGCTGGAGGTGGATTTTACCGTGCGCGGGCCGTTTTTG
GCAGAACCATCGGCATAGAATTCTTTATCCAGTTGCAGGGCCAATGTTTTGAGCGGCGCATCA
TTAAAGGCTGTTTTGGTCAGGTCGCCTTCTTTACACAGGGCCAGAGCATTTTCCACATCCTGGG
CTGATTTTAAGACTTTGGCGATATCAGCGCGGACCAGTTCGGATTTCACACTCAGGTCGGCCA
ATGGCTTCACCCCATTTTTCGACGCGGCAATTTGTTGGGATAAAACGGTAAGGAAGACATTTTT
TTCCCCCACGCTATAGGCACTCATCAAGCCTTGGACCAGTTGCAGGCTTTCTGTTTTGGGATGG
CCGTTACCGTCCAGCAGGTTCACCAGCAAGCGGGTGGCGGAGACGTTGGTGTCGTTGAGGATA
ATGTCAAACATGCCCAGCAGGGTGGTTTGCTGGGTGATCGCCTTAAGCGGATCATTTTCCAGT
TCTTCTTGTGCGCGTTTGGCGCGTTCATCGCGTTCTGTTTCCAGTTGCACCAGCATGGAAAGTT
TATGTTCTATGGACCCCAGATCAGCATGATGGCTGTTCAGGGCCGAAGCAAAGGCACGGTGCA
TTTCCCCGTTTTCGATCAGCGCTTGTTGATTGGCTGTGCCGCTTTGGGAGATTTTCCCAAGCAG
GTCTGACATGGCGGACAGGGCTTCGCTCTCTTTAACCTGATCGGATTTCAGTTGGGAAAATTC
GTCTGATTGCGCTTTGGAGTGCAGGGCCAGCTTTTCGACCAGTTCTTTATTTTTTTGCAGGATG
TCCTGATAAAAAGACAATCCGACCACAACAACAAACAGCAGGAAAGGTGGGAAGATAAAGGCA
GATAAGACCCCGGCAACTTCATGGGGCAGGAGGTAGAAGAAGTTCCCCCAGCCCATGTAAACC
GAAATGTAGCTTGCCCAAACGAATGTCCAAATCAGAGAAGGGACGATAAAACCCGTTTGAAAG
GCTTTGCCTTGAAGAAGATGCTTGATCATGTCGATTTACCCTCACGCATGAAAAGCGCGACCCA
AATTCGCGCTATAACCCTTTTCTAGACTTTATGCAATTACGCAGGGCAGTGCAATATTAAGAGG
CGGATTTTATTCACTTTTCGCACTGTTTTGAAGGACATCACGAATTTTCATATCCTCTTCCATAA
TGTGGTGCAAAATCCATTCTTTTAAATAAGCGGTGAGCTTATTGGCAAAAGGTTCCAGATCCTC
TTCATCCAGTAAAAGATATTCATCCATCAGCTCTTCAATTTTTACAAGGAAGGTGCGATGCTCT
TTAATATGAAGGGCAAATTCAGGATAGCCTGCCTGATGCATGGTTTTTTCTTCAAAAGAAAAAT
GTTCCTGCGTGTAATCAGACAGATTATTCAGTGTGTCTGTAATTTCTTGCGTACTGAGCTTTTG
CGAAAGATGGTCGTGAAGCGTATTAATCCACCCACAAAGTTGCCGATGTTGTTCATCAATGACT
TGTACGCCCAGTAGATATTCGTCTGTGAGTTGAATAAACACCTTATACCTCAATGGGTTGTGAT
CATCTGTGAGGGTAGAATAGGCAGTCTGTTTTGTTTAAGTCAAGTCTGGCTATCAAAGGTTGG
GCAAGCTATTGTAAGGGGGCATTGAGATGAAATGGGAAAGAATTTAAGATGGCTCTTTGGCAA
AGTCAGCTGATTACAAAGACTTCTGAGGAAATCACACACGCGCAATATTATATGAACAATAGTT
CAGATATTGCTGTCTGGTTTGTATTTTTTTTCCTGATTGCCCTGTTTGTTGCCTTGGGATATGCG
GTGCATCGTTTTTTGCCCCCTTCTGTTTCACCGAAACCTGAAGAAGAAATTGAAACAGATATCA

GCGAGGAAATAGAGGAGGAGGGGCCTTTGGTGCAGCCTCCCAAAATATCATTTGGGCATGTCT
GTGTTCCTGAGGGTGCAATGAGTGTGACATGGGTGAATTTGGCCGGAGAAAGCTGTGCGGCG
GTTGTGAAAAATATTACGACCAAAGAAGTAATTTTTGATGCGCCGAATTTTAATGGCGATACAA
TAAAGGAAATCACCGTCTCTCTCACTAAGCACCGCTTCATTGTTGAAGAGGCACATGTGCGCG
ATAGTGACGGGGATCAGGTAGTAATTGCTTTGGAACGATTTCAGGATAATGAAAACAGCTGGA
TGGCCTGGATCGAACTGCAAACACGCATTGACGGGGTATGATAATGCTTTCTGCGACTTATGC
TTTTTGGACTTTTTTCGGAACGGCCGTTTTGATTCAGGCCGTTGATGAAAAACATAATATTACC
GTGCGCCGTAAGGTGAAGGATAAAAAGAAACCGGACCTTTTACAAAAACAGGGGGCGCAAGGT
GCGGCGCAAGCTTTTCAACCGATGACTGAAAAAGAGCGCGAGATGCTTAAGCAGGTGCAGGCC
ATGGAAAATGACCTGAATACCTATCGTGAACAAAAGCTGACAGAAATGAATGGGGCCTTGGAA
GAACAAAAGAAAAGGGCGCTGGCCGAAGTCATGGACTGGTCGGTTGAAGAGAAAAAACGCCT
CGAAGAACGCTTTAATCAGGACTATCAAAGCGCATTGGCGGGGATGCGCAATGAGGTGGTGCG
TCAGATTGATGATGAGTTTGAACAAATCATTGTACATTTTGAAGAAATGGCTGTTGAAGACAAA
ACCATGGCGCAAAACCAGATTCGTGAATTTCTGGAGAAAAAGAAGAAAAAGCTTGTTTCCAGT
CTTGGTTGAGAATGCGTTTTATCGAAAAAGATAAAAACCCCTAAGATTGGTGAGGTTTTAAAGG
GGCGAAAAACACCGGAAAACTGCGGAAAACACCAGTGTTAATAATGACTTGCAACTGGCTTTT
TGGGCTTTTTAAACTTGCTTAAAAGTGAATGCTTATGTATGTTAGGTGCAAATAAACATCTAGA
TTTATCGGGGGTACCTGAATGTTATTACTTAAAGCGGAACAAGCGAGATCAGTTCCTGCACTTG
TTGGCAAGACATGGAAAGTCGGTGAAGTCACCAAAGCTGGTGGCGGCGCAAAAGGCTTGACCA
ACTGGCTGGTGTTGCACCCAGCAGGCTCTGGTGCGGGTTCCAAAACTGTTATGGTTAAAGTGA
CTGGTGCGGGCCAGCAATTGCCGTCTTTGGTGGGTAAGACTTTTACATTCGGTAAGTCTCCGG
TTCTGGGCAACGCTGGCATGAAGTACATGGCGCTCTATCCGGCCAAGGCTGGTTCAGCAGCCG
CTGGTGCTGCGGCAGCAGGTACAAAAGGTACGGTTGCGGTTAAGCTGGCAAACACAAAAGCAG
CAGCTCAGCTGCAAGCGTTCCAAGGTAAAGCTTTCGTAATCGGTAAAGCGCCGGTTATGGGCA
ATAACCTTGCAAATAACATGCTGGTGCTTGAACCTCTTAAAGGTGCGGGTGTTAAGACTGGTG
CGGCGGCTGCGGCCAAAGGTGGTGCGGCAGCGAGCAAAGGTTTGATGATCGGTAAGACGACA
ATCGGTTCTGCCGGTGTTGTAACAAAAGGCGCGGCTGCGGCAGCCCCAGTTGCGATTATCGGT
GCAGATGCGGCTGGTAATGCAGCAGCAACAACTGTTGCCAAAACGGCTGCGACAAAAGCGGTT
GCGACAAAAGCAGTAGCAACCAAAGCGGCAGCAGCGACAGCAGGTAAAGCAGCAGCTGGTAA
GGCTGCGGCAGCTTCTGCGGGTGCGGCAGCGGCCGGTACAGCAGCAAGCGGTACAATTTGGA
CTGGTACAGGTCTGAGCCTTGGTCTCGGCCTCGGTCTTGGTGCGTTGGGTCCGGTTTTGTTGA
CAGCAGCGGCTGCAACAGGCGGGTACTTCCTGTACAAGCGTAATAAAGACGCCGAAGAGTGTG
AAGCGGAAGAGTTGGATGCCACAGAAGCATTGATCAACCCGCCAGCACCAAGCGGTAATGTGG
CTCCGGCCTAAATCTAGGGGAAAAAAAGGAATTTAGCGGATGGTTGCGATGAATGGTGGTAAT
GCAGCAGCTTGTGTGGCAGGTGCCGGTGCTGGTAAAGGCACTGGTGCTGCTAAAGCTGGTGCT
GCGGGCGCTGCACGTCGTGAAATGGAACGCGAAGGTATGCGTAATGCAGGCGGTAAAGCCGG

TGCAAAAGGCATGGCTGGCAAAGGTGCAGGTGCCAAAGGTGGTGCTGCGCAGGCCGCTCGCG
GTGAAATGGAACGCGAAGGCATGCGTAATGCTGGCGGCAAGGCTGCGGGCAAAGGCATGGCT
GCAAAAGGTGGCGCTGCGAAAGGTGCTGCCGGTAAAGGTGTAGCGGCAAACGGCGGTAAGGC
TATGGCGGCCAAGGCAACAGCTAATGGTGCTGGTGCAACGGGTAAAGCTGCTGCAGCGACAAC
GGCAAAAGCTGGTGCAACCAAAGCAGCTACGGCGAAGGCGGCTGCGGCAACAACAGGCAAAG
CGGCAGCAACGAAGGCTGCTTCTGGCACTGGGGCTGCTGCGGCGACAGCCAAAACTGTTGGTT
TTGGTCTGGGGCTGAGCTTCTGGGCACCGGTTGCGGTTGCAGCTATTGGTGCTCTGGCGGTCT
ATGGTTATATGCGCAAGAAAAATGCGGGTATCGACCAGTCTGAAACAGACGCTGAAATTCAAG
AGGCACTCAGCTAAGGGTAGGCTGAGTTTTGGGGGCTGGCCGTTTATTTTGGCGGCCAGTCGG
AAGTAATATTTTAAGGAACCAATGGATATGGTTATGTCAACAGTTCAAAGTAATGGTGGGATGA
AGTCTCGCATCATGGCAGCGATGAGCTATCTCGGTATTTTGTGCTTTGTACCTCTGTTTTCTAA
CAAAGATGACGAGTTTGTCTATTTTCACGCGAAACAGGGCCTGGTTATTTGGGTCTGGGGCGT
TCTGGCTGTCTTTGCACTGCACCTGCCGGGTCTTGGCAAGTGGTTCTTCAGTGTGTCTGCCATG
GCAATCGTCATGTTTTCTCTGATCGGTTTTGTATCTGTGATGCTGAACCGCGCATGGAAGCTTC
CGTTTATTTATGGTGTTTCTACAAAGATTTAAATCTTGGTGAAGACCTGAATAACACAAGTCGC
TTCAGCTGTTTTTTATCGGCTGTAGCGACTTTTTAATGTAAGAAAAACGAATCGACTAAAGAAA
ATTATGTCAGATCACTCTTTCGAACGAGAACAACGTAACGCCTTATATTTCCTGTCGGCGATCT
GCATGGTCTTTATGACTTTGGCAATTGCTGTTCAGCCGTTGTTCTTGCGCAATGTTCTGGATAT
TTCTTTTGAAACAGCCGGTGCGGTAAATGCCAGCGTACAGGTTGTGACAGAGATCCTCGATCT
CGCCTTGATCTTCTATCTGGGCTATTTGTCGGATCGTTTTGGCCGGGTGCCGATTGCCACAATC
GGCTTCCTTGTTGCCGCCCTTGGTGCCTTGCTGGCACCTGCCAGTGCCCAAATCGGTGTCTGG
ACAGGGATCGGCGGTCTGGCATTCTATTATATCATGCGCATCATCATGTCCCTGGGCACAGGC
GCGGTCTGGCCGCAAATCTCTGCTTTGGCAGGGGATGGGACAGATTTTGATAATCGTCCTCGC
TTTATGGCGAATATGGCCTTTATGATGGCCTTGGGGGGAACCCTCGTTTACGCCATCTTGATGC
AGATGCCACAATATACAGGCTATATCGTTGTCATGGTTTTGACCGCAGGGGTGGCTTTGATCG
GGGCCTATGTTTCGCATCGTTGTCTGGTCGATATCGCGCCCAAGCTGGAAAAGAAAGCATTTC
CGTGGAAGCGTATTCTTGAGCATCTCAAAAAAGATGATCGCCTGCGTCTGAGCTTTGCCAGTG
CCTTTTTTGCCCGTGCTGACATGGTGTTCATCGGGCTGTTCCTGATGATGTGGTTTATCTACTT
TGCCGATCTGGTGAAAATCGAACAGGAAGTGGCCGCTGCGGAAGCTGGTTCTCTCATCGGTCT
GGTCGGTATTGTGGTTCTGGTTTCCCTTCCGGCATGGGGACGTGCCATTGAGGTCATGGGTCG
TGTGAAAACCCTGGCTCTTGGTATGGCCTTTTCCGGTGTTGGATTCGTTGGGTTGGGCTTCATC
GTCAATCCGTTTGAGTGGGAAATTGTCCCGTTTTTGGTTTTAGCTGCCTTGGGCCAGGCTGGCT
GCCTTGTGGCGCCGCAGGTTCTGACCATTGATTTGACGCCAAAGGATATTCGCGGTTCCATGC
TGGGCATGTTCAACGTGGTTGGCGGCATCGGTATTGTGATTTTTGTTCAGATTGGCGGCTTCCT
ATTTGACCATATTGGACCCCATGCCCCGTTTGTGCTAATCGGTGTGGCGAACTTCGCTGTTGTG
ACTTATGGCATTTGGGTGTTGCGCGGTGATGGAACGGACGAACAGATTTGTGAAGACACGCTG

GAAGATGGGGAAGACATCAACAAGGTGTTTGAACAGTAAGAAATTTTGATCGCAAATTGACGA
TCAGCATTTTTTTGCAAAGTTTCTTCTTTTATTTATGAAGGAAGAAACAAAATGAGAGGATGAC
AATTAATGCCTGTCGTGATTTTTGGCCTGGTGGCCATTTCATTGGGTCTTTGGGCGATGGCTGC
CTGGTGGTGGTCTGTAACCGAGCTAATGCGTGGCTTGGTGCCTATCATTCTCGTTATTTTGGGT
ATTGTCGCTGTTGCAGCTGGCCTGTCCAAAGTACGTGAAGACAAACCTGTCGATGACGAAGAC
CTACTTAACGGTGAAGGATAATTCACAGTGGAAGATCAAAAAGTATTATTGGATCTGGAAGAC
GAACTGGAAGACAACGTTCCAGAATATTGCAGCCTTGAAATCCGCAAATACCTTCTGTTTGTCG
GTTTGATCGCTGCCATGATCCTTGTTGGTACCTTCTGGTATATCAACTATTACAAAGAAGGCAT
CGGTGTTGAAACCGCTGCTG<u>TGGCACTGGGCAATGACACT</u>GCTGTGACTGAAACGTCAAAGCA
GTTTGCCCAGCCCACGGGTCCCAAGGCGCAGCTGGTGAACTGGTTCCCGCGCCAACCGGGTG
GGGCTGCACCGGGCTTTGGTGTGTCTGTTCCTTCTATGGGGAACCCGCTGATCCCGGCTGCA<u>A</u>
<u>TGCCGATGGGTCAAGCTGG</u>TATGCCGGTTGCTATGCCTGTGGCGATGCCAGGTGGCAAGAAAG
CACCTAAGGCGCTCAAGGGTGGTTTTGCCTCTGTTGCTGATGTGATCCACGATAGTGTGGTCA
ATATTTCAGCTGTTCGTGGGGTCGGTCCACAAGCACCACAAAATAAGCCTAATCCGGCTATGG
GTAATATTTTCAGTAATCCGTTTTCCGGGCGTTCTTTTGAGAATATCGGTTCCGGCGTAATTGT
GCGCGACGACGGATACATTGTAACGAACTATCACATCATCCGGGACGCTGGTTCCGTCACCGT
AAATGTCTTTACGGGTGATTTGACAGATCGTTATCCTGCAACCGTTATCAAGCTCGATGAAGTG
CTTGATTTGGCGTTGCTGAAAATCGAACCGAAAAAACCACTGACAGCAGGTATTCTGGCCAAC
AGTGATCTGGTCAAGGTTGCCGATGAGGTGATCGCTGTGGGCAGCCCCTTTGGTCTCAGCCAA
ACGGTCAGCCGTGGCATTATCTCTGCCAAGCGTAAATCATTGACCATTGAAAATATTACGCACG
CGAACCTGTTGCAGACAGATGCGGCCATTAACCAGGGGAACTCCGGTGGCCCGCTTGTCGGTG
CAATGGGTCAGGTCGTCGGGATTAATACGGCGATCTATACGCCAACAGGCTCTTTTGCCGGGA
TTGGTTTTGCTGTTCCCAGTAATCAG<u>GTTCGTACTTTCTTGCTCGA</u>ACAAATCAATGGCCTGCC
GACACGCGTTAAAAAACCGATGATGCAGACTGTGGCGATGCCGCAAGGGGGCGCGATGGCAC
CGCCGATTGCAGCCAATGCCAAGCCGCCGCATACGGATGGCCGTGAAATGATGGATTGTCAGA
CTTGTCACCAGATCACAGGTGGTGGTGTTGCGGCACAGACTGTGGCTTTGACCAAGCGTCA<u>GA</u>
<u>TGCGTCAAGGCCCACCGA</u>TTGCAGCCAATGCCAAAAGCCCGCATGGCGATGAGCGTGATCAAA
TGAACTGTGCCATGTGTCACCAGATTAATGGGGGCCCGAAAACCGCAAATGGTAAGCGTCCGG
GCATGCAGGTTGTGGCTTTGACCAAACGTCAGATGCGCCAAGGCCCGCCGATTGCACCGAATG
CACGTAACCCGCATAATGATGAACGTGCGCAGATGAATTGTGCCATGTGTCACCAATTCACAG
GCAACGGCCAGCAAGCCGCAGCAGGTGGTCAGCCTGCCATGCAAGCTGTGGCCTTGACCAAAC
GTCAGATGCGTCAAGGTCCCCCGATTGCGGCCAATGCGCGTAACCCGCATAATGATGAACGTG
CCAAGATGAATTGTGCGATGTGTCACCAGATCAATGGCGGTGGACAGCCCGCAGGTCGTCCGG
TTGCCATGACACAGCCCACTGGGGCACCGCCAATTCCGGCCAATGCCAAGCCACCCCATACAG
ATGGGCGTGAGACCATGAACTGTGCAACTTGTCACCAGATCATTGGGGCAGGTGGAGCAACGC
CAGCGGCTTTTAACTATTCCTTTGCAGGTCCGAACAATCCTTCGGCCTGAATGTAGCAGCCCC

GCAATTCGGTGGTATGACAAAGGAAATGAATAAAGCTACATTGCCGGCCCGCGATTTTTTTATC
CAGGGCGCTAGTGTTCTGCCAATCGATTCACCGTTGGTGCAGCACAATGGTCCCAGTCCGGGG
CGAGGCGTCTTTGTAAATAAAGTTATGCCAAATTCACCAGCGGATATGGCTGGGATTAAAATCA
GTGACATTATCCTGCGTGTGGATGGGCGTCGTGTGAATTCTCCGTTAGAGATGACGCAAGCGA
TGGACAAAATCCTTGTCGGGGATCCGGTGCGTTTGACGGTGATGCATAAAGGGCGTCGTGCTG
ATCTTGAATTGATCAAGAAAAAAGCTCCAACCCTGAAAGATATGCAGACCATGCAGCAAGCTG
CGGTTCAGCAAAAGCCGAACACACCAACCTTGCGCAAAAAAGCACAGGCACAGATCAAACAAC
CTGTTCCGACAGAATTTAACTGGCGTGGCATTGAGGTTGAAAACTTCCGCCGTCCCACCCCGG
CAGACTCTCCGATGGGTAAACAACCCGGTGGGGCCATTGTGGGGGATGTGACACGGGGCTCC
CCTGCTGAACGTGCGGGGGTCATGCGCAATGATGTGATTCTTGAGGTCAATGCCCGTTCTGCC
AAAAATGCGAAGAAATTCAATAAAGCGATTAAAAAAGCGCGAGAAGAGCGTATGATCGTTTTG
AGAATTGATCGTGCAGGCCAAGAAAAGTTCCTTGTGTTGCGCTGATCAGCGTTAAACCATAAG
CGAGCAGTTGGGATTTAGTCATGGCAAACGATCCGAAAGATCAGAAAGAGGCCCCTGAACAGG
AGCAGTTTGATATGTTGGCAGGCAGTGAGCCGGAGCCAAAGCCCAAAGCTGCGCCTAAGAAGC
CTCCGGCAAAGAAAACTGCCGAGGTAAGGGACGAGCCGAAAGAGGATTCTGAAACGGAAAGC
AAGCCTGCATCTCCAAAAGCGCCGACAAAGCCCAAGGCAACGCCGCGTAAAACGGCAACGGC
GACCAAGGCAGCTGCCAAAAAGGTTGAAGATGATAAAGCTGCGCAAGATAAGCCAGCGGCGA
AGAAAGCGACAACAAGATCGACTGTAAAACCACAAGCAACTTCAAAAAAAGAAGAAGAGGCTG
AGGTCGTCGAAAAAAAGTCTGCTGCCAAAAAAGAAACTGCGAAGACGCCAACAAAGGCAAGGA
CAGCCTCTTCAACAGTGAAGTCTACAACGACAAAGAAAACAACCAAAACCACAACAACTAAGG
CGAAGCCAAAACCAAAGGCCGAGCCTGTTGAGGAAAAGAAAGAAGCTGTTGTTGAAGAAAAAA
AGCCTGAACCTATTTCACCGCTTTTGATGGATGCCTTTAATGTCTCGCCTTGGGCCGGGGCACT
CATGGGGCAGGGTGCACAGCCCATTATTCAGCCTGATGAAATTATCAAGGTGCCGGAAGCTGA
ACTGATTGTGGAAGAAAAGCCGCAAATTACACCTGAGACCCCCCCCGCAGATCCGGTCAATTC
TGTTAGTGCAGAACCTATTCATGTGGAAACTGTCAAGGCCCCTGTTGCAGAAGAAAAAACGGT
TGAAAAACAGCCTGAGCCCACAAAAGTTGAAGCCAAGGCCGAAGAAAATAAACCGGAAAAAAC
AGTAGAGCCTGCAAAAGTACAAGAACCAGCCAAGCCTGTGTTCAAAAATGCCAGTGGCGCAAC
AATTGATTCGCCCTGGAAGACGTTCAACAATGAACCTGCACAGCAAACCAGTGACAGCGAAAC
CATTACCTATAAGGCACACGCTAAACCAGTAGAGCCTGCTGTTGTCATTCGCAGTTCGACAACG
CCAATACCTGCGCCTGCTCAGTCTTCTCCCCCTATGGAACCTGTAGCAACAGTCAAACGCCGCC
TTAAACGTGGTGAACGTAAGGCCATGCGGGCTGAAAATATTGAACATTGGCTACATGAAAATG
GGGATGGTGAGATTCCAACACGAACCCCTTCGCAGCCAACTGTAGGGGCTAAACAGCCAGCAG
CCCCCGTTGAAAAAACGTCTGAGCCTGCACAAAAGCCGAAAGCATCAATTCCATGGCGTGAAC
CAGAAGCGGTTATTCCGCCAGAACTGGCCAAGGTGCAGATTGAACAGGCAGCTGCCAAGCCGC
GTGTATCAAAACGTAAAAAGCCGACAGCGTGGGATGGGAATCCGGCGACAGAAACAAAACGAT
TATCTGAACGTCTGGCAGAAGAACAGCCGGAAAGACAGCCGCAGGATATCGCGGTTGAAGGTA

TGGCTATCGGTATTATCAATGCCCTTGGTGATGGGGTGGAGGCTGTTGTCGATACGGCAAAAG
GGGCTGTGGCCTCAATCGGGGAGCAGCCGACACGTCATTTGGATAAGGATGAAGTCTTAGTTG
AAAACATTGCCCGAGGCACGGTTGATGCCTTGGGAAGTGGGGTGGAAGCCATTGTGGATGCAG
GCACCTGTGCGGTCAAAGGGATCGGCATGGGGATTGATTGCGGCGTGCAATCTGTGAAAGGC
GGCGTAACCCGGATAAAAAATGCCGGAAGCGGTGCTGCACACGGTTTTGTGGATGGAATCAAA
AAGCCTACATCTTCAAAGGATTGAGTCTGAATTATGTTAGACAAAAAAGGGTCTAACTGGTATC
AAACATGGGGGTCCGGCCCAGCGTCGGATTTCTGAAAAAATAGGAACTGTATAATGGTCACTA
AAGCAACAGAAACACAACAAGAAGCTGAGAAAACAGAAACAACTGACTCAGCTGAAAAAACAG
CGACTACGCCAAAAGCGGCTGACAAACCGGCTGCGGCAAAAGCTGAACCTGCGGAAGATAAAT
CTGTTGAAACAAAGACTGAAGCAGCTCCAAAGAAAGCTTCTGAAACTGAAGCTGAGAATGCAG
ATGCACCCAATGGTCAGGAAATCCCGGTTGAGGATCTGGTCAACGGTGTGATCAATGTTCTTG
GTGACGGTCTGGAAGCGATCATCAATTCAAGTTCATCTGCGCGTCAGAATGTTCTCCCTCATGC
CAAAAGCAGCTTCAATGAAATGGTAAGCGGTGTGAAGGATGGCTTTACTGATCTGCGTGGATA
TGTAAATGACAAGCTCTCTTCCAATAAAGATGCTTCTGTTGAGGAAGAAATCGACGCTGCTGTT
AGTGGGGATAACAAAGCGGACGAAAAGGCAGAGAGCGCTCCGCAAAGTTAACAGGTTTATTAA
AGGTTTGAACATATGAGCAGTACACAAGACAGCTGGAACACTGGCAACGACACAACAAATGCA
GGCGGTGAAAATGAACCACTGCTGATCGGCTTTGATTTCGGCACTTCTTATACTGCGGTGATGT
CAAACCGTGGGCATCAGCAGGTTTTCCGTTCTGTTGTGGGTTATCCCAAAGATATGATCGGGG
TAAAATTGCTGGGTGAACCTTATGTGGTGGGCGATGCAGCCTTTGAAAAACGTTCTTTCGTTGA
TCTGCGCAGCCCGCTGAAAGACGGTGTTCTGCGTGAATATGTGGAACGCGATATGGAAGTCGC
GCGTGATTTCGTTGACCATACCATCAATGCCATTGCCAAAGAGGGCGATGAAGTTGCTGTGAT
TGTGGGTGTTCCAGCACGTGCGTCAAACACCAACAAGGACCTGATGGTTCAGGTCTTTGAAGA
TTTTGTTGATACGGTTCAGGTGATTTCTGAACCGTTCCTCGTGGCTTATGGTCGTGGTTCGCTG
GTGAACAGCCTGGTCATTGATATCGGGGCAGGGACTGTTGACATCTGTGCGCTTAAAGGCGCC
ATGCCGGGTCAAAAATCCCAGATTACGATTAATAAGGCCGGTAATTTTATTGACGAACAGATCG
AAGCCTTGATCATGCAGGCCCACCCGGAAGTTCAGATGAATATCCATATGGCTCAAGCCATTA
AGGAAAAACATGGTTTTGTCGGTACGGCTGCGGAACCCGCCATGGCTGAATTGCGTGTAGAAG
GTAAACCGGCACAGTTCGATATTACCCGCGAAGTCAGCGCGGCGTGCGAAATGATCGTTCCGG
GTATCATCGAAGGTGTGGAAAAACTGATCCAGACCTGTTCACCAGAAGATCAGGCCACCATGT
TGAATAATATCTTCCTGGCTGGCGGCGGCTCGCGCATTGGCGGTCTGGACAAGATGATTGCAG
AACGTCTGGTTAGCTTTGGCGATGTGCAGGTGTCCCGCGTTTCTGATCCCACCTATGCTGTGG
CGTCCGGTGCGTTGAAACTGGCACAAGAGCTGCCACCGCAATATTGGGACCAATTGGGCAGCG
TGACAGACAACTAAGTCTGAGCTGTCTTACAGGTCATTTGGAGAATAGAACGTGGCAAAACTG
ATCATATATTTGATCTTGGCGATTGCTATCTTGTTATTTGCGCTGCAAAACAATGTGCCGGTGG
TGATTGATTTGATCTTTTTGCCCCCTGTGCAGATGCCGATGATTTTGGTCCTTGCTCTGGCCTT
TTTCAGTGGCTTCATTGCGGCACTCTTTAGTGTGGCGCGCATAACAATGAAAAAGAAAAACCG

GGAACAGATCGAATATCATCCCAAATGAGATGATGTTTGCTTTGTTTCCAAATGACCTTTGAGA
CGACTTTAAAGACTTAGGATTTTAGGGAATGAAATACGATAAATGCGGTAAGTGCATAAAAACC
ATTGGCTGGGTCGGCTTGGTGGTCAATACCGTGCTGACTTTTCTCAAGGCTTTTGTCGGGCTG
GTTTCCGGTTCCCACGCCCTCTTGGCCGATTCCCTGTATTCCTTTAAAGATGTGGTCAGCTCTC
TTCTCGTCATTGTCGGCGGCAAGGTGTCTGAACAGACGTTGGATAAGGAACACCCGTACGGCC
ACGGCAAGATCGAATTTGTCCTCTCCCTCTTTGTCAGTGTGGTCTTTCTCGTCGTCACCGGCTT
CTTGCTGGTTTACGCCATCTCCATGCTGTTTGGCGATAATATCCACGAAGCCCCGCACCTAATC
GCGCTTTGGACCGCTGTGCTTTGTGCGGTTGTCAATGTGGTCATGTATGCCTATTCCAAATGTG
CGGCTAATGAGAGCAACAGCCCGCTAATCAAGACCCTGTCCAAACATCATCACGCCGATGCCT
TTTCTTCAAGCGCGGTTGCGGTGGGGATCGTTGGGGCACATTACCTCAATATGCCCTGGATTG
ATACGGGTGTGGCTGTGGTGGAAACCCTGCACCTGATGCATTTGGGCGGCCATGTCTTTAAAG
ATTCTGTCATGGGCCTGATGGACCGTCAGATCGACATGCCGAAACGCAAAATCCTCACCAATC
TGGTGCTGGGTGTGGAAGGTGTGAAGCAGCTCAAAGAAATGCGCACACGCTATATCGGTCAGA
ACATCTCTGCGGAAATCACCATCGGGGTGGATGAAAACTGCACCGTCGATGAGGCCACGGCCA
TTGCCGATAAGGTGAAAGACACCATTGTCCATACGGTGGCACGCATTGGCGCGATTCAGGTCA
AGGCTGAAGCACAAGGCAGCAAGGAAAGCACGGCTGCTGAAGAAGCCGCAGATGCCATGAGC
TTGCTGGATGAGGGTCCCGCACCGCAAGGTGGAACGGCTTAAAAAGCCAAGTAGTGAATAGAC
GAAATTAAAGTGACAACCAACAGGTTGAGAAAGACATGAGCGAGAAAGAAAACGTAAAGAAAC
TGGCCTTTTGGGGCGATCGGGGATTTTGCGCAAGTCCGTGGCAAGGTTTGGCCATTGGCTTTA
CGGCTCTGGTCTTCTTGACCTTTGCCTGGGGCTTTGTCGTTCTGGATGGCTTTAAGGAAGACGA
TCATTTTGATCGTTTCCACGATCTTTCCATCCCTGAATTCCTCTTCCGTGATGCTGCGGGTGCC
CCGCAAGCCATTGCCGATGCCTTGGCACCGGGGATTGTCGGGATCAGTGGTTCTCAACCCAAT
ATGCCGATGATGGCGTCTGGTTCTATCGTGAGTGCTGAAGGCCATGTACTGACGGTTCTTCAT
CCGTTGAAAAACCTTGATCAGCTTTATGTCCATGTACGCACGGTTGAAGGCATTACACCCTTTA
AGGCCGAAGTGATTGAGACCAATGCCAATCATAATCTGGCCTTGTTGAAGATTACATCGCCAG
AACGTTTTCAATATTTCACGCTGGCAAATACCTCAAACCTGAAGGTCAAAACAACAGTTGTTGC
CTTGGGCCAAACCCGCAATGGCAATATCGTTATTAACCAAGGCCAAGTGCAGACGCTGGATAC
ATCCTTGAAAGTCAATGGTCGCCCGATGAAAGCATTGGCGGCGACCGACGCGGTCTTTTCCTG
GCAGCAAACAGGTGGCCCGCTGGTCAATACCCGTGGTGAAATTGTCGGTGTCAACGTTGCCTT
TAAGGGGCCAAACGGTGTTGTTGATGGCTTCATGATCCCCAGCTATGTGGTCATGACCCATTTT
GGAAATATTGCGAAATTCAAGGTGGGCCAAGACGTTGTGGTTCCGGTGATGTGGAAACCTAAA
TCCCAAGTCGGTAATAACGCCCAAGTGACTGCCCCACCGATGTGGCAGATTACAGCAACACCG
AACCCGCAAGTTCAAACGTTGAACCCGAGCCAAACCACGTCTAACACCTTTGGCATGAATGTG
GTTGCCAATGGGGTGAACCACGCCATGACCGATCTGGAGCATCTGGGCGGCACGCGTATTTTG
GGTTTTCCGCTGAAAGATATTATCGGCTTGGCCCTCTTGGGTCTGGGGGCTGGCTTGATCAGC
GGGATGATGACTATGGGCGGCGGCATCTTGCATGTGGCCGGGATGATGATTGTCTTTGGTTAT

GGGATTTACCTCATTCGTCCGGTGGCTTATCTGACCAACTTGTTTATCTTCGGGGCCGCAGCAC
AGCGTAACCTGAAATCCGGTCTCGTCATGTGGGATACGGTGAAAAAGCTCCTGCCTTGGGCGG
TAATTGGTATGGTTGGCGGTTACTTTATCGGTAACTATATCGGGGATGGTGGCATTGCCTTCTT
GCTCGGTGCCTTCGCTGCGATTATGACCCTGAAGGGCTTGCATGAAATTTTTGTCCCTGTTCAG
GAAAATATTCTGGTCAAAACAGGTGATGATAAAGAAGATAAAAACGTCATTCATGAAGGTATTG
ATGATGACGATTATATCGACGAGCTTCTGGCCGAAGAAAAGACAAGCCGAAGATCGGGTCCT
GGGCACTGGATATCAAGGATGTCTTGCTTGGTCTGCCCATCGGTTTGATCAGTGGTATTCTGG
GTATTTCCGGTGGTGTTCTGGCCGTTCCGCTGCAACGCTTCTTTAAAGGCCAAGCCATTCAAAA
CGCCATTGCCAACAGCTCCATCGTTGTCTTCTGGGCCTCGGCCATGGGGGCATTGGTTGCCTT
TATCCACGGCACGAGCGCGGGCCTGATTGATTGGGAAACACCGCTGATCATGACCATGATCAT
GGTGCCGGGTGCCTTTGCTGGTGGTGCGTTGGGTGCACGTTTGATGAAGGTTCTGCCTTCCAT
CTTCCTGAAATGGTTCTACACCATCATCATGGCGGCCATCGCGGTTCGTATCCTCTTCTTAAGC
TAAGGAGAGGCTGATGACACGCAATGTCATATTGGCTGGTTTTACGTTAGGGGCGGTTTTGCT
TGTTTTACTTGTTGTGTTTTTTGTCGATAGCTTCGGCTTTGGCAAGAAGGCGGCGGGTGATCAG
GCTTCTGCAGCGACCTCTTCGTCGCTTGCTTCTCCTGCGCCTCAAGGCTCGACCTCTGTACCCT
CCATGGCGGCGGTTCCGCCTGTGGCAGCCTATCAGAGCCCGTCTCTTCAGGGTAATGTGGGGG
TGTCCCCGGTGCAAGGGGCGGTTCAGCCGATGAGCCCGAACGTGAACATTCAGTCACCGCAAC
AAGCTCAGGCACAGCCTGCCCCGGTTCAGCCGGATATGGCGGCGCAATCCAGCCCGGCCATCC
AGTTTCAAAAGCCGTTGAGCCAGTTGATCCAGCCGGAAAAAGCGGCTGAGGTGCCGCCAAACT
ATATCCCCAAGACGGTGGAGCTGTTTGAAGCGCACTGGCAGGGCCTTGATAGCCGCGCCTGGA
CCGATGAGCTGCGCCGCAAACTTAAATATCCCAAGGGGCTGACGGGTATTCTGGTCGGTGAAG
TCACGCTGAACGCCGCCGAAGCCGGTATTCTCGCAGGTGATATCATCAATAAGGTGGGCGATG
TCAAAGTCAGCTCCCTTGAAGAATTCCAGATGGCGACCCGTGAAGTGCGCACCCAGACCAATG
TGAAGCTGCGCCTCATGCGTCCGGGTAAGAAAAAGCAAGACGGCCGCTTCCCCATGCGCACGC
TCACCCTGGTCATGATGGGCCAGCCGGATTTGGGTATGGCACAGGTGGAAGGGGCACCGATG
ATCCTGCCCGGTGATCCGCGCCCGCACCCGCACCGCGGAGTCTGTACCAATTGCCACACAGTG
GGTGATGGTTTTGAGCTGAGCCCGGACCCGGATTTGGTCTCTGTCCCGCCGCCCGTTATCGAT
CATGCCACTGTGGTTAAGGGTATTCGCCCGCACCGCGACCGTGGCCCCTGTGAAGCCTGTCAT
TTGATTCGTAAAGACCAAAAAGGAAAGAACCCATGAGCATTATCAAAGGCGACCTGGTTGGGC
GTTCGGAAGAATACGCTCAATATACCGCGATCATCTTAAAACGTTTGGGCACCAAACTGGTTG
GGGGCTTCCACGACCTGTTTACCATCGATGATGAAACGCGCATGGAATATTTCCGTGACAAGG
CCATCAATCTGGCGAAAGCCGGCAAGCACCAGCGTGCCGGCCAATTGCTGGAACAGCTTTATA
AATCCAACCCGGAAGACGGCGAAGTCATGCTGCATCTGGGCGTGTGCTACCTTAAACTCGGCC
ATCGCAATGAAGGGATCGAGCTTCTGGAAAAAGCCAGCGATGAACATAAGGACGATATCAAAC
TCGCCACCGTGCTCGGCCTGTCTTACATTCAGAATGAAGATTTTGAAAAAGCCATCCCTTTGTT
GGAAAAAGTCATTGAAGATACGCCGAACTCGGCCAATATCCTCTATCGTCTGGGTGTGGCTTA

CGACAATACAGGTAACTATCAGCGCGCGGTGGAATGTTTCCTCAGTGCGCTTGAGATCAAGCC
GGATGAAGCGCGTATCCACCGCAGCGTGGGGTATGCCTTTGAACAGATGGATGATCACGAAGC
AGCCATGGCGCATTTCAAACGTGCCAACGAGCTCGGCGGTGGGGAGTAATCGCTTTTAAAAGC
GAAGGTTAGTGTGAACCATGGTGAATTTCTTTAATACCGGCAATCAGGCCCCGTCTCAGGAAC
TGCAACGGCTCAAACGCTCCGAAGCGATGCTGGCGCAGCTCTATCGTGATGAGACGGAACCGC
GCTTTTCCGTGCCGTCCATGAAAGGGGTCAAAACCCTGTTTCTGGTCTCGGTGCTGGGGATTTT
GCTGTTTTCCGGCACACTATTTTACAAGTTCAACGACTTCATTATTTTCCGTGAAGACGTGCTG
GCCAAGGCCGGGAACCTGCAATCAGCCTTACAACGGCGCAAAGACTTGTTCTCCAACCTGGTC
AACCTGACCCTGAACCATGCGTCTCTTGAACATTCCATTTTCTCCTATACCGCCAAAATGCGCA
CCGAGATCATCAAGAAAACTGGTGATGTGCTGCCACCCGAAGCGGTGAAAGATGCCTTGGGGA
AAAACAAGGATCTGGCCAAAGGCTTAGGTGCCTTGGGTGTGGGCGAAGCCAATGGGGCAGAC
GGGCTGGATCTTAATGACATGGGGGCATCGCTTGGTCGCTTGCTTGCCGTTGTAGAAAAATAT
CCGGATATTAAATCCGCCCAGACTTATACGGAAGCCATGGTGGCTTTGGTGCAGATGGAAGAC
CTGATTACCCAGCGTCGTATGGATTATAACGAGTCCTTGCGGATTTATAATTCAGCCATCTCCA
AATTCCCCTGGAAGATTTTGGCGGATTTCACCAACTTCCCGCGCTTTGAATATTTCAACGAGAA
AACCATCACCGACAGCGCCCCGAAACTGGGTCTGGAAACCTATCGCCCGTTGGTTCCTTTCAT
CGATGAGGGAGGCGGACACTAATGTGGACAATGATCGCACGGGTGGGCACCTTAATGTCCTTA
GGCGCCGCCCTGATGGAACTATACAATTCGCGCCATCGCGAAGTGAACTCAAGTAGGATTTAT
AACTCTACGGAAGCGGCCCGTTTTTTGGGTGTTGAACGTAAGGAAGTGATCCGACTTTTAGAA
CAGGGACGCATGCGCGGGCGCCTTGTCAATGGCAATTACCGCGTTCCCGGTAGCAGCATAATT
GAGTATTTGAGCCATGATTCCTAATAAATGTAAGCAATGTAAAGAAGAAGTCGTCTGGTGGGC
CATTGTCGTCAATATCTGCCAAATGACCTATAAGGGCCTGTTGGGCGCCATGACCGGATCTGT
GGCTTTGGTGGCAGACTCGCTGCACTCTGGTGCGGACGTAATCGCCTCTATCGTGACCATGTT
GTGTGTGAAAATCTCCAAGCGCAAGGCGTCTGATAAATATCCTTATGGCTTCGGGAACATCCA
GTTTATCTCTTCGTCCATCGTCGGGATCATTCTGATTTTGGGGGCGATCTACCTGATGTATGAA
TCGATCCTCAAGATCATTGAAGGCAATATTGAAGCGCCAAGCTTCCTTGCCGTGCTCGGTGCC
GGTATGTCTGTTGTGGTCAATGAGCTGATGTATCGCTACCAGCATTGTGTGGGCAAAGAAAAC
AACAGCCCGGCAATTATCGCGAACGCGTGGGACAACCGTTCGGATGCGCTGTCTTCTGTCGGG
GTGTTGATCGGTATTTTGTTTGCGGTTCTTGGCTTCCCCATCGCTGACGTTGTGGCCGCCATGG
TCGTGGCGATCCTGGTGGCACGCATCGGGATTGAGCTGAACATTGATGCCATTGACGGCTTGA
TGGATACTTCTGTTGAAATGGACGTTCTTAAAGATGTTTACAATATTGCCGCCAACGTGCCGAA
TATTGAAGAGGTCCGTCACCTGCGCGGTCGTAACGTGGGCGAAGACATCCATCTTGATATCTC
CATCGGGGTGAGTGGCTCGCTTAAGGTCTATGAGAGTGATCTTATTGCTCAGGCCCTGAAAGA
ACGCATCTATGCCGAAGTCCGTCACGTCACCGACGTGCAGATTGCCGTTGTGAATAGTTAAAA
GAAAGCGCAAGAGGTCTAGACATGAAGGCGGCTTAACAAAAAAACATACTGGTCCGGAATTCTCGA
AACTGGTCATAATTGTTAATTCCCTAAGAAGATTGATAAAAAATTCAAAATATTCCCTCTAGTGTTTT

CATATGA

>*mamAB-2* (bold region: SH1_v1_0290 ~ SH1_v1_0310)

CACTAGCCCTGCGGAGTCGCCTTGTGTTAATCTTTTCTTAACTATTTTGAAAAAGGGCGATGAAGCTC
GAAATTGTCTGCTGTTCTTGGCAGGCTTTTTTGTAACCCATGCGCACCCATGCAGGTAAGACAGCACA
ATCAGGGGACCGACG**ATGGACTTCAACCAGAAACCGAATTACTCTCAGGAAATCAGTTCCAGCA**
CACCCGGCGCGATCCTTTTTCTTGTGGATCAGTCGCGCTCCATGAACAAGCCTTTTGGCACCAA
CGCAAGTGGCCAGCCGGTCAAGCGTGCTGAAGTCGTGGCCGAAGCGCTCAACAACACATTGGC
CGAACTGGTCAATCGCTGTACCCGCGATGAAGGGGTGTCTGATTATTTTGAAGTGGGTGTGAT
CGGTTATGGCCGAAATTCCCGCCCGGAATTCTGCTGGGAAGGTCCACTGAAAGGACGCCGTAT
GGTGTCGATTTCCGAAGTGGCGAAACATGCCCATGTGGAACAGAAAACCATTGAGACCGAAGT
GCGTGGTACCATCGTTAATGAAACGGTTTCTGTTTCAAGTTGGCTCTCCCCGGTTGCGGGGGA
AAGCACACCGATGAACGGGGCCATCAATATGGCACGCCAGACCCTGGAAGAATGGATTTATCG
CAACCCGAAATGTTTCCCGCCCATCGTCATCAACATCACCGATGGGATGGCAAATGATGTGTC
ATCTGCTAACGAGTTGATCATGAGCACCAAGCGCCTAACAGACCTGACGACCACAGACGGTCA
AGTGCTGCTGATCAATTGCCATATCACAGATGAGAATGACACCACCGTGACTTTCCCCTGGTCC
AAGATGGAACTGCCAGACGAAGATTATGCCCGCATCCTGTTTGAAATGTCCTCAGACATGCCG
GAACGCTATAAGGCCATCATCTGCGAAATATTTGATCGCGACCAGTCCCAAACCCCAATCGTAA
AAGGTATGGCGTTTAACGCAGATGCCACAGCCCTCGTCAAACTGCTCGACATCGGCACAAGAC
AAGCCTTCGTCATTAATGCCGCATCTCACGCGGCGGAGTAGAATAAGAATATGCAAGAGACTG
CTGCTGCCACTGTGACAAAAACAGTGACAGAAATTGATGTGCGCGGCCAAACCATGGCTAAGC
CAAAAAATGATGTGATCAACGGGGCTACCCAGGCCGTTGTGCGCCAGAATGAAGATCGTTTTC
GTGTTGAACAGAATGACGGGCGTGTCCTGATGGCGGTGGCCGATGGAGCTGGGTCATCGGGG
ATGTATTGTGGGGCCTGGGCGGAAAAGCTGTGCTCCCGACTGCCTGAAACGCCGCTGTCTTCC
TATGATGATCTGAATGGCTGGATTGACGGTTTTTGGGAAGAGTTTTCCACTTTTGCCAAGAAGC
AATGTGCCAATAATCATGGACAGCATAACAAGCTGGTAAAAGAAGGCTCCTTTGCCACACTGG
TGGCAAGCTGGCTGGATCAACAAGATGATCGCCTGACATTGGATTGTCTGAATTATGGCGATA
GCACACTCTATGTGTTTGAGGAAATGGGCAATGAGGTCATGTTGACAGCTGCCCTTCCCGGTA
ATCTCGCCAGTCAGGAAGCGGACCCTCATTTGCTCAATTGGAAAGACTTGCCCAAGGCCGAAC
AGGTGCAGGTCTTAAGCCATGAGATTAAAAATGATGCCATGGTTGTGCTGGCTTCTGATGGCA
TGGGGCTGTTTGTGATTTTGCGTTATTTATCCTATCTGGCGCAAAAACAGGAAACGGCCTATAG
TGATGTGGCGAGAAACTTCATGTCAGAATATCGCCACTTGTTACAAAATGGCACAAGCCCGGT
TGCAAATTTTGTCCGCACCCATCAGGCAGAGCCGGTGTCGGACTTTAAAGGTGAGCTTCTGGA
TCTTTATCATGCCTTGGGAACTGCGGAAGGTTTTCAAGGATATATTGCTGAGCGTTATGAAGAG
GGGTTGCTCCCCAATGATGATTCAACGCTGGTGATGACCATGATTAAAAAACGCGAAGAGAGC
ATTGCGCAGAACTAACGCCTTGTCAGGCGTACCTTTTTAAGGGGAAGAAAGGACTAAAATGG

**CATATCCAATTATTACTGAATATAAAAACGCCCTGCGCAATTTTGAGGGCCGCTTTGCAAGCCT
GAAACCCAAGCCGTTTTTTGATGGTAAGGGAGAACCTGTTTTTTGGGCAGGGAACTTTGCCGT
GGTATTCAAAGCCTATGTGGAAGGCCGTTCAGAACCTGTTGCACTCAAATGTTTTATTAATGAT
CTGCCGGATTTGGAAAAGCGCCACCGTGCCATGTCTTTGACCTTTGCCAAGTTAAAAGCGCGC
TATCTGATTGATGTGGATTTCCTTAATGACGAGCTTTATGTGACCTCGAGCATTGGGGGGAATC
GCGATTACCCGGTTGTGGTCATGCCCTGGGTGGAAGGCGAAACATTAGGAGCCGTGATTGAGC
GCCAATGTGTTAAAAACAATCGTCGTGGGCTTGTGGCTATTACCAAAGCCTGGGCGAATTTATC
GCTGGATATGCTTGGCAAAAAAATTGCCCATGGGGATTTGAAACATGACAACGTGTTGGTCAC
ACCTGACGGGCAGTTGCGCCTGATTGATTATGATTCCCTTTTTGTCCCGGCCATGGCGAAAATG
GATTCCATTGCCATTGGCGGGCCGAGCTATCAGCACCCAAAGCGTGCTTTTTATCATTTTGACA
AGACACTGGACCATTTTTCCATGTTGGTGTTTGTCTTATCCTTGCGTGCCCTTGTGATTGAGCC
AGGGCTTTATCAAAAATATAACACCGGACAAAATCTGATTTTTACCGGCGAAGACTTTATTTCC
GGGGGACGTTCAGAACTGTTTATGCATTTGAAACGCAGTGCAGACCCAGTGGTACGTGAATGG
ACGGACTTGCTTATTAAGGTGATGCAATCTAAATCTATTGCGGTACCGCGTATTGAACGCGTGT
TGAAATTAGCCCGCAAAACAGAAATTTAGATTTTTTTCATGTCCAAAATTGATCTTGAAGTCAA
ACATAGCCTGAAACAGGAAGAGGCCGCCAGCCGTCTGGAAGCTTTCTTAAAAGAGCAGGGCAA
ATATAACAAACAGTTGAGCAATGCCGTCTTTGAACGTGAGGGCCGATTGTTTCGTTTTACGGCA
AAAGTCAAGGGATTCAAGATCAAAGGAGCCGTGGCCGCACTTGAACATTCTATCAAGGCGCAA
ATTGTCCTGCCCCTGACAGCACGTCCGTTTAAAGGTAGTGCAGAACAGATTTTACGTGAGCAAT
TGCAAAAAGCAATGAGTTGATTATACTTTTGTATAAACCTAAAGTATCATTTTTGGCACCCCCC
CCGCAAATTGTTAATGATTGATTAACAATAGGTTATGCGGTATTATGAATCGTCTAAGTTGAGT
CTTAATAGTTGTATTTCGATTTTTGATTCGAGTTAGCAAGTGTGTCATCAGCTGAACATAGGTG
TTGAATATGTCAAACGTGTATCACCTTCAGAACCTTGGGAAAGGCTCTACTGAATGGAATGCAT
GGCGGGAGTCTGACAAAAATGTGACCCCGGACTTATCCCATGCCGAAATCAAAGCGGTGAACC
TTGCCCGAATCGACTTGCAAGGTGCATTGCTGACCAAAGCCAACCTGAAACAAACCAATTTGA
GTGGCGGTGATTTATCCGGGGCCAATATGGCCGGAGCCAATATGGAAGGGATTTCACTGGCGG
GCTCTAACATGGAACGGGCCAATTGTGCCGGGGCCTATATGCATAAGGCCAACTTAGCAGGCA
GCAAATTACGCTGGGCAAACCTGTCTGGTTCTTCTATCGTTGGCAAAAGCAACATGTCCGGGG
CTGACCTGACCGGGGCCAATTTATGCGGTGCTCGCCTGATTGGGGTCAACCTGTCTGGGGCAA
ACCTGACGGGGGCGAATCTGGCCGGGGCTGACTTCAGAGATGCGGACCTGACCGGGGCTGAT
TTCACCAATGCCGATACCAGCGGTGCCTTTTTTGGTGGGGCAGATCTGCGCGGCACCATGCTG
GAACAATATTACGATCTGTTTGGCGCAGACGATGAACCAGCAGCCCCAGAAGAAGAATATGTC
GAAGAGGTTGTTGAAGAAGAACCTGTTATCGAAGAAGTCCTTCTGGAAGAAGTGCCTGAGCCA
GAACCGATTATTGAACCAGAACCAGAGCCCGAACCGGAACCAATGCCGGAACCAGAACCAGAG
CCTCAACTGGCTGATATTCTGGAACCGCCTGCGCCGGAACCTGTCGTTGAAGATGACTTTGAC
GAGAATGCATATAATGTTTATGAGACAAAGGAATGTGCCATTGTCACCCTTTGTTTGCCCAAGT**

TTAAAGAAAAAAGCACGCGTGATGAACATGACTTCCTTGACCTGTTGCGTCGCTATAACCGCTA
TTTCTTAAATACAGAAGAAAATGTTGCCATGGCGGCACGTGGGGATGCTTTCTTCGGGGCATTT
GAAAACCCGAATACGGCGCTCAGTTGTGCGCGGGGCTATCTCAACATTTTGCGCGATATGAAA
ACCGATGCCTTTGTCGGGGTGAACTGGGGCAGCGTGACTTCTTGCGGGTCAGCCGAAAGCGAT
ACCAAGGATTTGATTATCAATTCCATCACACCGATGGCACGTTTGGTTCCACTGGCCGAACCGG
GGGAAGTCTTGATGCTGGATGAACTTTATAGACGTCCGGAAATCAAGAAAGACTTGTTCAGTTT
TGAAAAAGTATCGCGTAAATGGACCAAGACCTTTAATCAGGATGGGCCCAGCTTCGAAGTGCT
GTGTTATTCTGTCAGCGAAGCCAAATCTGAATAGATTCTCTATGAGAAAATGCGAAAAAATCCC
CTCTTTTTATGAGGGGATTTTGCATTGCAGCGAGGGTTTGTTGCAAAAAAGTCGCTGCATACGT
GTATATGGGCATGCTTGTGGGTGACTTTGCTAATTTATGCTTTATAATCGCGGAACTCTCAATT
AAAGTGCTGCCTCGTGGTCGGTTTTTGAATAATTATAAACTAAGGGTTCTTCGTTGAAATACAT
CCCTCATAGTGTCGCTGAATGGGTTATGGCTCTTGGCATTGCTTTCAGCTTGGGTATTTTCTTT
GTTGCCGTGGTGGAAGATAACCCTTGGCAGGATCATTCCTATGGTGATGCGCCACCGATTACT
CTTGGAACACCTGCCCCGCATACCGATGGTAAAGAGAAAATGACATGTTCAACCTGTCATGCG
ATTCTCCCACCGGACCCCAACCAAAGCAAAGATTTTCGTATTCCGATTGCCGTTGGTGCACCCT
CACCACACGGCGATGAGCGTGACCAACAACCTTGTAGCAACTGTCACCGCTATGTGAACAGCC
TGCAACAAGATGTGGGTGGTAGTGGTGTTGCCCAAGCTGTCACCGCTGCCATGGCGATGCCGC
CTCAAGGCGAAGTGAAGAAAAAACCGTCTTTACGTTTCCCGCCCAAGGCCAAGCCTTTGGATA
AAGAAGCTCATGAAATTTTCACGTTTTTCCGCTTTCAGGGAAAGGTGACACGCATTTTCCCCAA
AAATCCCAAGCTTGACCCACAAGCGAACATCGTGGCTCTGGTCGATAACGGGATTACACAACC
CATGTGGGTCGATCTCGCCCCGAACTGGTTCCTGAAATCAGAAGGATGCCGTGTTTTCAAAGG
CATGTTTATCAAGGGCCAAGCAGCAGCCGAAGACCCCAAAGATAGAACCGAGTTGGCTTATGC
CACAACACTCGGTGTAAATGGTAAATCCTGTTTCCTGCGTAATTCCCACTTGAGGGGACTATGG
GACCCAATGGCAATGATGGGGAAATAAGGCAATGATGTTATTGAATAATTTAGAAAGCAAATT
CCAGAACCATCAATGGAACGGTATTTATTTGCTATCGGCCATTTCAACCTTGATTGGGGCCTTG
GCGGTTTCTGTACAGCCTTTGCTGTTGGATCAGATTTTCAATATTCCCTTTGAAAAAGAAGGTG
AAATCAACGCCGACATTCAGGTAGTCGCCGAAATGGTTTCCATCCTCTGTGTCGGTTATTTCAG
TTACAAGTCTGATTTTATTGGTCGTGTCCCCATTATTTTCTATGCCTTTCTCTTTATTGCTGTCG
GTGCCTTCCTCACGCCGATGAGTTATGAGTTGGGTGTGTATCTGGGTGTTGGGGGGCTGTTTG
CCTTTTACTTTGCCCGTGTTCTGGTGACGTTGGGCTCTGATACCGTTCAGGTGCAGCTTCTTAC
TCTTGTAGGGGATCTGTCAGATTTTAAAAACCGCCCTAAATTGATGACAAATACAGTCTTCATG
GTGGTTTTTGGCGGTACAATCCTAACTGCTATCGTCATGCAGATTGCCGAATATGAATATGGCA
TTGAACTGATTACCTTTAGCCTCGTCCTTTTTGGAGTCTTTGGGGCATGGGTCACGAAATACTC
TCTCCATGATGTTGCAGAATTTTGTGCCGGGGAAGAAAAGCGCCACCCCTTGCTCCGCGTTTG
GGACTTGGTGTCCAATGACCCTCGCATGCAACTGGCTGTGGCTGCGGCTTTTTACACCCGTAT
GGATTTGGTTGTGGTCAGCCTGTTTTATTCCCTATGGTGTATTTCCGTTGCCGATATCATTGGC

GTAACCCGCGTTTATGCCACCGCACATGCCGCGACCATGGTTGGGGTCATGGGGGTCGGTGTT
CTGGCCTCAATCCCATTTTGGCAGCATTTGATTGAACGTCACAGTCGCATCACGGCCATTGGTG
CCTCTTTGTCTATTGCGGCTATGGGCTATGTCTGGATCAGCTTGTTTAAAAACCCCTATGACTG
GGGGATTGCCCTGCCGCTGCTCTTGATCGGGATCGGTCATGGCGGGGCGACGGTCACATTGAA
AGTTTTAACGGTTGATATTGCCCCAAAACCCTTACTTGGTGCTGTTCTTGGTATGATTTATCTG
GCCGGCAGTGTCGGCATTATCATGCTGGTACAAAGTGGCGGTTATTATTTCGATGCCGTTGGC
CCACGTGCTCCCTTTGTTTTGATGGCCAGTGGCAAGCTCATGGTGGTGATCTTTGCCAGTTGGC
TGGTCATGCATCAGGTTGAAGAAGATAAAGACCATGTACTGACCAAGAAAATCCAGATCAGCT
GGCGCCCCCTGATTTTCCTCACCTCCGCCCTGCCTTTTGCCTGGTTGCTGGGCCGAATGCTGTT
GGGGGGCTATCTCTGGGGGGTGGATCATGAAAATATTCCGGTGGGCTTTATCAACCGATATCT
CGGTGACTGGGCCTTTACCTTCCTGATCCTGTCTTTGGCAATTACACCTTTTGCCGAACTGACC
AAGATTAAAGCGGTTCATAAATACCGCCGTATGATCGGGCTCTATGCCTTCTTCTATACCATGT
TGCACTTCATTGTATATGTGTCACTGGAATGGACCTTCGATCTCGATCACATGCTGGCAGATGC
CTATAAACGTCCGTTCATCTTCCTCGGGATTGTAGCCTTCTGTATCATCACACCACTGGCGGTA
ACATCGCTCAAGTCTGTGCGCAACAAGATGGACCCGAAAAACTGGCGTCGTTTACATAAAGGG
GCCTATATTCTCAATATCATCGTTGCGTGGCACTTTATTCTGGCGGCAAACAAGGAAAATGGTG
AACCTTATATCTATGCAGCCTTGATTGCGATCCTTCTGGGCTATCGTGTCTATGAGAATATTCA
GAAACACAAGCGCCGTGCAAATCGCAAACCGCGCCGTAAACGCCGCGTGAAAAAGAAAAAATC
AGCTGATGGCGCAACCGGGGATGCCCCCATCGCTGAAGCAGCATCTGAATAAGCCATAAAAAA
AGGGCTGAGGTCATAAAAAACCTCAGCCCTTTTTCTTTCTCTATAAAAACCTTTCTACTCCGCC
GCGTGAGATGCGGTATTAATGACGAAGGCTTGTCTTGTGCCGATGTCGAGCAGTTTGACGAGG
GCTGTGGCATCTGCGTTAAACGCCATACCTTTTACGATTGGGGTTTGAGACTGGTCGCGATCA
AATATTTCGCAGATGATGGCCTTATAGCGTTCCGGCATGTCTGAGGACATTTCAAACAGGATGC
GGGCATAATCTTCGTCTGGCAGTTCCATCTTGGACCAGGGGAAAGTCACGGTGGTGTCATTCT
CATCTGTGATATGGCAGTTGATCAGCAGCACTTGACCGTCTGTGGTCGTCAGGTCTGTCAGGC
GCTTGGTGCTCATGATCAACTCGTTAGCAGATGACACATCATTTGCCATCCCATCGGTGATGTT
GATGACGATGGGCGGGAAACATTTCGGGTTGCGATAAATCCATTCTTCCAGGGTCTGGCGTGC
CATATTGATGGCCCCGTTCATCGGTGTGCTTTCCCCGGCCACAGGCGAGAGCCAACTTGAGAC
AGAAACCGTTTCATTAACGATGGTACCACGCACTTCGGTCTCAATGGTTTTCTGTTCCACATGG
GCATGTTTCGCCACTTCGGAAATCGACACCATACGGCGTCCTTTCAGTGGACCTTCCCAGCAG
AATTCCGGGCGGGAATTTCGGCCATAACCGATCACACCCACTTCAAAATAATCAGACACCCCTT
CATCGCGGGTACAGCGATTGACCAGTTCGGCCAATGTGTTGTTGAGCGCTTCGGCCACGACTT
CAGCACGCTTGACCGGCTGGCCGCTTGCGTTGGTGCCAAAAGGCTTGTTCATGGAGCGCGACT
GATCCACAAGAAAAAGGATCGCGCCGGGTGTGCTGGAACTGATTTCCTGAGAGTAATTCGGTT
TCTGGTTGAAGTCCATCGTCGGTCCCCTGATTGTGCTGTCTTACCTGCATGGGTGCGCATGGG
TTACAAAAAAGCCTGCCAAAAACAGCAGACAATTTCGAGCTTCATCGCCCTTTTTCAAAATAGT

TAAGAAAAGATTAACACAAGGCGACTCCGCAGGGCTAGTGGTATTTTCACACTTGCCACAAATGACACCTTTGCAATTTAGACGCAGTTAAAGCTGAGAGTAGTCACTCAAACTCAGCGGCTTCCAGTACGGCGAACTTATCTGGACTCCCGATCATATCGGGAATAACAAGAGAGTGTTAGAACCACTTAAAGCCATTATCCTTTGGCTTTTCCAAGGGGACTTTGACGACGATATCGTGACATTTGATGCAGCGCCCCGCAGGTGGGTGGGGCATTTCAGAGTTGGGCGGTAAAGGTGGCCCATCTTGCGCACATTCTTGGAAATCTCTTCCAACGCAGCCCCCACCGGGGTCTTATATTGATTGCCCGGTCCCGGGCCGCCAACATAGAGGTGACATTGCGTACAGGCCCCCACATAAGGGTGTGGCATACGCTGCCCGGCCTTAATGGCTGGAATATGCTTCATGGTCATATAGCGATATTCCGTGTTGGGTGGGGTCAGGCGCTTTTCAATTTCCGCCCCGATACCTTCTTCAAAAGGTGTCTGATGCTGACCCAGGAACAAGCCCTGAATAGTGCGTTTGGTCAGGGCTGTTAGTTCCGTGCCATTGACCACGACAAAGGAGAACCCACAGACGATCAGCACGGCGATAAAGACAGAGCCTCGTCGTCCTTCAAAGAGGGTCAGGATATGTTTGAATATCCTCATGACTTTTAGCCGAGGCGGTTTGACCATAGGGCAAAGCCTTCGTCGTTGCGTAGGCGACAAGTGATGCCGTCAACGACAATATTCTTGGCGTAAAGTAAGCCGTTGGGGTCTTGTTTATCGAACTTGTAGGCGATGCCGCGCACGTTTTTGCCTTTGGAGAGGCGACAGCCCAAGTGATTGAGGAACCAGTTGGGGCCGATGGAGACTTCTTTGGCCACACCCGGTCCGGTATCTACCCAGATATGAACTTGCCCCCAGCCGATGGTATCGCCCCGGCTGAGCATACGCAGCACATTGCCTTGATATTTCACCGTCGGGGCTTTGTTCAGGGCCTGCATCTGGCCCATTTGCGCATCCGGGCGGTTCCAGGCAAGCTCGCCGCCTGTGCCTTGTTGGGTCGTCTGACCTTTGGGGGTGGCGAGCATCTGGTTGGACGGGGTCATTTCCTGGGTCTGATTTTGCACCTGTTCGCGTTTGATATCGCGCAAACGTTCCTGGGCATCAGGCTCATAGAGCCCGGAAGAGACCAGAGCCCCGATGACCATGCCCACAAAGGCAAGGCCACCAATCCCTAAAACCAGATGTTCTGACTTCATCACTCGCCCTCTTGCGCTTTCTTTTAACTATTCACAACGGCAATCTGCACGTCGGTGACGTGACGGACTTCGGCATAGATGCGTTCTTTCAGGGCCTGAGCAATAAGATCACTCTCATAGACCTTAAGCGAGCCACTCACCCCGATGGAGATATCAAGATGGATGTCTTCGCCCACGTTACGACCGCGCAGGTGACGGACCTCTTCAATATTCGGCACGTTGGCGGCAATATTGTAAACATCTTTAAGAACGTCCATTTCAACAGAAGTATCCATCAAGCCGTCAATGGCATCAATGTTCAGCTCAATCCCGATGCGTGCCACCAGGATCGCCACGACCATGGCGGCCACAACGTCAGCGATGGGGAAGCCAAGAACCGCAAACAAAATACCGATCAACACCCCGACAGAAGACAGCGCATCCGAACGGTTGTCCCACGCGTTGGCAATAATCGCCGGGCTGTTGTTTTCTTTCCCCACACAATGCTGGTAGCGATACATCAGCTCATTGACCACAACAGACATACCCGCACCGAGCACGGCAAGGAAGCTTGGCGCTTCAATATTGCCTTCGATGATCTTGAGGATGGATTCATACATCAGGTAGATCGCCCCCAAAATCAGAATGATCCCGACGATGGACGAAGAGATAAACTGGATGTTCCCGAAGCCATAAGGATATTTATCAGACGCCTTGCGCTTGGAGATTTTCACACAGAGCATGGTCACGATAGAGGCGATTACGTCCGCACCGGAGTGCAGGGAGTCTGCCACCAAAGCCACAGATCCGGTCATGGCGCCCAACAGGCCCTTATAGGTCATTTGGCAGATATTGACGACAATGGCCCACCAGACGACTTCTTCTTTACATTGCTTACATTTATTAGGAATCATGGCTCAAATACTCAATTATGCTGCTACCGGGAACGCGGTAATTGCCATTGACAAGGCGCCCGCGCATGCGTCCCTGT

**TCTAAAAGTCGGATCACTTCCTTACGTTCCACCCCCAAAAAACGGGCCGCTTCCGTAGAGTTAT
AAATCCTACTTGAGTTCACTTCGCGATGGCGCGAATTGTATAGTTCCATCAGGGCGGCGCCTA
AGGACATTAAGGTGCCCACCCGTGCGATCATGGTCCACATTAGTGTCCGCCTCCCTCGTCAAT
GAAAGGAACCAGCGGGCGATAGGTTTCCAGACCCAGCTTCGGTGCGCTGTCGGTGATGGTTTT
CTCGTTGAAATATTCAAAGCGCGGGAAGTTGGTGAAATCCGCCAAAATCTTCCAGGGGAATTT
GGAAATCGCTGAATTATAAATGCGCAAGGACTCGTTATAATCCATACGACGCTGGGTAATCAG
GTCTTCCATCTGTACCAAAGCGACCATGGCTTCGGTGTAGGTCTGGGCCGATTTAATATCCGG
ATATTTTTCCACAACCGCAAGCAAGCGACCAAGAGACGCTCCCATGTCATTAAGGTCCAGCCC
ATCTGCCCCATTGGCTTCGCCCACACCCAAAGCGCCTAAGCCCTTGGCCAGGTCCTTGTTTTTC
CCCAAGGCATCTTTCACCGCTTCTGGCGGCAACACATCACCAGTTTTCTTGATGATTTCCGTGC
GCATCTTGGCGGTATAGGAGAAAATGGAATGTTCAAGCGACGCATGGTTCAGCGTCAGGTTGA
CCAGGTTGGAGAACAAGTCTTTGCGCCGTTGTAAGGCTGATTGCAGGTTCCCGGCCTTGGCCA
GCACGTCTTCACGGAAAATAATGAAGTCGTTGAACTTGTAAAATAGTGTGCCGGAAAACAGCA
AAATCCCCAGTACCGAGACCAGAAACAGGGTTTTCACCCCTTTCATGGACGGCACGGAAAAGC
GCGGTTCCGTCTCATCACGATAGAGCTGCGCCAGCATCGCTTCGGAGCGTTTGAGCCGTTGCA
GTTCCTGAGACGGGGCCTGATTGCCGGTATTAAAGAAATTCACCATGGTTCACACTAACCTTCG
CTTTTAAAAGCGATTACTCCCCACCGCCGAGCTCGTTGGCACGTTTGAAATGCGCCATGGCTG
CTTCGTGATCATCCATCTGTTCAAAGGCATACCCCACGCTGCGGTGGATACGCGCTTCATCCG
GCTTGATCTCAAGCGCACTGAGGAAACATTCCACCGCGCGCTGATAGTTACCTGTATTGTCGTA
AGCCACACCCAGACGATAGAGGATATTGGCCGAGTTCGGCGTATCTTCAATGACTTTTTCCAAC
AAAGGGATGGCTTTTTCAAAATCTTCATTCTGAATGTAAGACAGGCCGAGCACGGTGGCGAGT
TTGATATCGTCCTTATGTTCATCGCTGGCTTTTTCCAGAAGCTCGATCCCTTCATTGCGATGGC
CGAGTTTAAGGTAGCACACGCCCAGATGCAGCATGACTTCGCCGTCTTCCGGGTTGGATTTAT
AAAGCTGTTCCAGCAATTGGCCGGCACGCTGGTGCTTGCCGGCTTTCGCCAGATTGATGGCCT
TGTCACGGAAATATTCCATGCGCGTTTCATCATCGATGGTAAACAGGTCGTGGAAGCCCCCAA
CCAGTTTGGTGCCCAAACGTTTTAAGATGATCGCGGTATATTGAGCGTATTCTTCCGAACGCCC
AACCAGGTCGCCTTTGATAATGCTCATGGGTTCTTTCCTTTTTGGTCTTTACGAATCAAATGAC
AGGCTTCACAGGGGCCACGGTCGCGGTGCGGGCGAATACCCTTAACCACAGTGGCATGATCGA
TAACGGGCGGCGGGACAGAGACCAAATCCGGGTCCGGGCTCAGCTCAAAACCATCACCCACTG
TGTGGCAATTGGTACAGACTCCGCGGTGCGGGTGCGGGCGCGGATCACCGGGCAGGATCATC
GGTGCCCCTTCCACCTGTGCCATACCCAAATCCGGCTGGCCCATCATGACCAGGGTGAGCGTG
CGCATGGGGAAGCGGCCGTCTTGCTTTTTCTTACCCGGACGCATGAGGCGCAGCTTCACATTG
GTCTGGGTGCGCACTTCACGGGTCGCCATCTGGAATTCTTCAAGGGAGCTGACTTTGACATCG
CCCACCTTATTGATGATATCACCTGCGAGAATACCGGCTTCGGCGGCGTTCAGCGTGACTTCA
CCGACCAGAATACCCGTCAGCCCCTTGGGATATTTAAGTTTGCGGCGCAGCTCATCGGTCCAG
GCGCGGCTATCAAGGCCCTGCCAGTGCGCTTCAAACAGCTCCACCGTCTTGGGGATATAGTTT**

GGCGGCACCTCAGCCGCTTTTTCCGGCTGGATCAACTGGCTCAACGGCTTTTGAAACTGGATG
GCCGGGCTGGATTGCGCCGCCATATCCGGCTGAACCGGGGCAGGCTGTGCCTGAGCTTGTTGC
GGTGACTGAATGTTCACGTTCGGGCTCATCGGCTGAACCGCCCCTTGCACCGGGGACACCCCC
ACATTACCCTGAAGAGACGGGCTCTGATAGGCTGCCACAGGCGGAACCGCCGCCATGGAGGG
TACAGAGGTCGAGCCTTGAGGCGCAGGAGAAGCAAGCGACGAAGAGGTCGCTGCAGAAGCCT
GATCACCCGCCGCCTTCTTGCCAAAGCCGAAGCTGTCGACAAAAAACACAACAAGCAAAACAA
GCAAAACTGCCCCTAACGTAAAACCAGCCAATATGACATTGCGTGTCATCTTACTGGCCGCCTA
AGCCCATAGTGTTCTGTGCTTTTTGCCACCAGGACTGTTGCTGTTGCACAGGCTGGCGCATTTG
CTGCATCTGTTGAACAGGCATTTGTTGCATTTGCTGTTGCATGGCCATCGGTTGAGCCGTTGCG
CCCATGCTAGGGCCCCAAGCCACAGGGGCAGCGGCAGGCGTCATTTGGCGAACTTGCGGGGC
TGCAGGGGCTTGAACTTTGGGTTTAGCAACCTTAAAGCCAACCACATCCTGAAAGTGAGAGAC
AACCACGTGAGCGGGAACAATAAAGCCTTCAACCGTATTGTTTGCCCCGCTGAATGCCATGTT
GACACCAACGATTTCACCACGATTGTTGATCAGCGGACCACCTGTTTGCTGCCAAGAGAAGAT
CGCATTGGTCGCAGCCAGTGATTTCATTGCCACACCACCTGCGTTGATGGTGTTGTTCAGGGA
TTGGATAGACCCGTTGTTGATCAGGATAGAGCCACTGTTGGTTTGACCCATGGCGATTACAGC
AGAGTTCACACCAAGACCAGCCGTATCGGCCAGTGTGAAATATTGGAAACGTTCCGGTGTCAG
AATTTTCAGGACAACCAGGTTATGTGCATCATGGGATTTCGCCACTTGGGCTTCATAACGTTTG
ATACCTTGCAGGGTACGTACATGAACGTCAATGTCTGTCAGACCCTTGATTGGATGAAGGGCT
GTCAGAACATGACCTTTCGGGCTGACCAAGGCACCAGAAGACACCAGCGGCATATTGGCGCCA
GCGGCACTAATACCAACGATACCTGGTGCCAAAGAATTTGCCAATTCCTGCGGGGAGGGGCCA
CCAGCATCACGAAACAGGAATTCAGGCACGCTCAGGTGCTGATATTTTTCAAAATGGTCATCAT
GTTTGTATCCGTTAAGGATCACAAAGCCCCAGACAAAGGTCAGGATGACCATGGAGGCAAAAC
CAAAGGCCAGACCACGCCACGGAGACGAACAGAAACCTTTTGTCCCGATCAAAGAACTATGTT
CAAGTTTTTCGCTTAGTTTCATCTTTTAATACTCTACTGGCGTGTCTGTTTAAGTGTCAGTTGTT
CGGTTGTACGCATCATTTTTAAGCCGTTCCACCTTGCGGTGCGGGACCCTCATCCAGCAAGCTC
ATGGCATCTGCGGCTTCTTCAGCAGCCGTGCTTTCCTTGCTGCCTTGTGCTTCAGCCTTGACCT
GAATCGCGCCAATGCGTGCCACCGTATGGACAATGGTGTCTTTCACCTTATCGGCAATGGCCG
TGGCCTCATCGACGGTGCAGTTTTCATCCACCCCGATGGTGATTTCCGCAGAGATGTTCTGACC
GATATAGCGTGTGCGCATTTCTTTGAGCTGCTTCACACCTTCCACACCCAGCACCAGATTGGTG
AGGATTTTGCGTTTCGGCATGTCGATCTGACGGTCCATCAGGCCCATGACAGAATCTTTAAAGA
CATGGCCGCCCAAATGCATCAGGTGCAGGGTTTCCACCACAGCCACACCCGTATCAATCCAGG
GCATATTGAGGTAATGTGCCCCAACGATCCCCACCGCAACCGCGCTTGAAGAAAAGGCATCGG
CGTGATGATGTTTGGACAGGGTCTTGATTAGCGGGCTGTTGCTCTCATTAGCCGCACATTTGG
AATAGGCATACATGACCACATTGACAACCGCACAAAGCACAGCGGTCCAAAGCGCGATTAGGT
GCGGGGCTTCGTGGATATTATCGCCAAACAGCATGGAGATGGCGTAAACCAGCAAGAAGCCGG
TGACGACGAGAAAGACCACACTGACAAAGAGGGAGAGGACAAATTCGATCTTGCCGTGGCCGT

ACGGGTGTTCCTTATCCAACGTCTGTTCAGACACCTTGCCGCCGACAATGACGAGAAGAGAGC
TGACCACATCTTTAAAGGAATACAGGGAATCGGCCAAGAGGGCGTGGGAACCGGAAACCAGCC
CGACAAAAGCCTTGAGAAAAGTCAGCACGGTATTGACCACCAAGCCGACCCAGCCAATGGTTT
TTATGCACTTACCGCATTTATCGTATTTCATTCCCTAAAATCCTAAGTCTTTAAAGTCGTCTCAA
AGGTCATTTGGAAACAAAGCAAACATCATCTCATTTGGGATGATATTCGATCTGTTCCCGGTTT
TTCTTTTTCATTGTTATGCGCGCCACACTAAAGAGTGCCGCAATGAAGCCACTGAAAAAGGCCA
GAGCAAGGACCAAAATCATCGGCATCTGCACAGGGGGCAAAAAGATCAAATCAATCACCACCG
GCACATTGTTTTGCAGCGCAAATAACAAGATAGCAATCGCCAAGATCAAATATATGATCAGTTT
TGCCACGTTCTATTCTCCAACCAATATCTTCACGAGCCTTGCACAGGGGCGGGTTGCGAAGTCT
GGTTTTCTTTGCGGATTTGCTGGATGATTTCAATGTCATCAATTTTTGGTTTGGTGCGCAGGTC
ATAGAGCTTTTGATACATAAAGGCACTCATGTCCGCGATCCCTTCGTGAATACCTTCACAGACC
ATAACATAGCCTGCCTCGGCATGGGGGGCGAGTTTTTGATGTGCTGTCATGCAGCCATCAATG
ACCACTTCCAATCCATCGCCGAGAACATTCACGACACCGTTAAACAAATCCTCAACAGGAATTT
CCTGTTTTGCTTCTTCAGATGGACTCGGGGTCGCGACAGACATTGGGGCCTGTTCTGTTGCAA
GGGCAGCAGTGGCCTGATGTTGCGGGGCGTCCGAGTTCATAAATTCAATCCGACTGTATATAT
TACAAGTTCAAAATCTTTGAGTGATGGAATACCAGTGTAGTCTGATTTTGTCTAATAAAAAAGG
TCCCTCAACTATCTGAATGTACAGTTTTCTTAGAAGTTATTAATGAGAATGAATTGCAATAAAT
AATTTTAAAAAAGACTTTGTATGCTATCGACTTGAGATTATTATGGTATTCAAGATTGTAAGGG
AATTTTCAAGCCTGAATTCTATTGCGAGATCAATAGCTTGATTGTCAGTCCTTATGATTGGTGC
TAGAGTTATTTTAGCGATTCTAAGTTAGAGTCGTTCTGAATTTGATTTTAAACATATGGAAGTG
TAGGAATCATGGCGTTTCAATTAGCCCCTTATTTGGCTCAAAGCGTTCCCGGTGTGGGGGCTTT
GGGCGCGATTGTCGGTGGATCGGGTGCACTGGCAAAGAATCTTCAAAAACATAAAGCCGGTGA
AATGGACACAAACGAAGTTGTTGTCGATACAGCGAAAGAAGCTGCTGGCGCAGGTGTTGCGAC
GGCTGTAAGTGCTTTTACCGTTGGTGTTGTTGGTGGCGGTCTGGCGGTTTCAGTTGGGACGGC
CTTTGTTGCAGCGGTTGCAGGCAAGTATGTTTGGGATCGTGGTATGGAATATATCGAAGGCGA
TAAGGATTTTGCCCCGATTCTCGATGAAGAAGCCCTGAAATAAGAGCTGCTTTTATAAAAGAGT
ATTAAAAAGGGTCGCTGAACATCAGCGGCCCTTTTTTTGTGGGGCCACAAATGTTGGGAGTGT
CGTAAATAAAAAAGGCCCGAACGAGTCGGACCTTTTTCTTTATTCTTTATAACGAACGCTGTGT
CAGAGAGTTTACACAGTGTCCGCCTTGATGGATGGTTCATCCAGAACAACGTTGGAACGGTGT
TCGCTGTATTTTTTGTAAGCGAACCAACCGCCAGCACCAACGCCAGCCAACAAGATAACCGGA
CCCCAGATGCCAAGACCCAGACCAAGGCCGAGACTTAAGCTTTTAGAGCAACCAGCCGCTGTA
CCAACAGCTGCTTTGGTTGTTGTCGAAGTACCTGTTGCAGCAACCGCATTTGTGCCCGCTTTTG
CTGTTGCGATACCTTTGCCTGCACCCGCAGCTGCCAGTTTGGCTGTGCCTGCTTTGGCTGCAG
CGCCGGCTTTGATCTTTGCCGCTGTTGCTGCTTTGGCTTTGGCCGCAGCAATCGCTTTTTGGTC
GGCGATGGTTTCAAGACTGTCAGACATGGTCTGAATTTCTTGCGGCGTCATGCCACGAACGAG
TTCATTTAATGGTAACATGGGTCCCCCGAAAAAAAACATACGCCCTTATGCGCGAATAACCCTAAA

TTTATTAGAGTAATTTAAAAAAGTCAAGGGAACGAGTGAGTTATAATTACAGTTGAGAGTTGTTCTT
ATTTGCTGACAAAAGCTCACGGCACATGACTTTTGAATGGCGCACCACAATATAAGTCAAGGGCATG
AGGTGAATGAT
