# Supplementary material for: Magnetosome Gene Duplication as an Important Driver in the Evolution of Magnetotaxis in the Alphaproteobacteria
Source: mSystems. 2019 Oct 29;4(5):e00315-19. doi: 10.1128/mSystems.00315-19 (PMC6819731; doi:10.1128/mSystems.00315-19)
Supplement: TABLE S2 [file mSystems.00315-19-st002.docx]

|  | SH-1 | PR-1 | MV-1 | QH-2 | MSR-1 | BB-1 | SP-1 | AMB-1 | MS-1 | SO-1 | XM-1 | ME-1 |
| --- | --- | --- | --- | --- | --- | --- | --- | --- | --- | --- | --- | --- |
| Mms6 | 41.3 | 40.0 | 38.1 | - | 41.5 | 42.5 | - | 38.1 | 45.9 | 44.2 | - | 42.1 |
|  |  |  |  |  |  |  |  |  |  | 50.5 |  |  |
| MmsF | - | - | 52.3 | - | - | 64.1 | - | 66.3 | 76.1 | 66.3 | - | 66.3 |
| Mms36 | - | - | - | - | - | - | - | - | - | - | - | - |
| Mms48 | - | - | - | - | - | - | - | - | - | - | - | - |
